# Resolving endogenous protein organization in cells with nanometer resolution

**DOI:** 10.1038/s41467-026-76146-7

**Published:** 2026-07-29

**Authors:** Janna Eilts, Marvin Jungblut, Dominic A. Helmerich, Stefan Sachs, Christian Werner, Cristian-Alexandru Bogaciu, Ali H. Shaib, Silvio O. Rizzoli, Philip Kollmannsberger, Sören Doose, Markus Sauer

**Affiliations:** 1 Department of Biotechnology and Biophysics, Biocenter, University of Würzburg, Würzburg, Germany; 2 Rudolf Virchow Center, Research Center for Integrative and Translational Bioimaging, University of Würzburg, Würzburg, Germany; 3 Department of Neuro- and Sensory Physiology, University Medical Center Göttingen, Göttingen, Germany

**Keywords:** Nanoscale biophysics, Single-molecule biophysics

## Abstract

Latest refinements in super-resolution microscopy achieved spatial resolutions in the one nanometer range. However, improvement of localization precision advanced faster than labeling methods impeding the translation of such high resolutions to cells. Hence, imaging of the nano-architecture of endogenous multiprotein complexes remains challenging. Here we introduce an expansion microscopy (ExM) method using double-homogenized hydrogels that enables *direct* stochastic optical reconstruction microscopy (*d*STORM) of 7-8-fold expanded immunolabeled samples. The ~4-fold increase in labeling density resolves the 8 nm spacing between neighboring α-tubulin molecules in microtubules, the polyhedral lattice of clathrin-coated pits, and provides evidence for the 8 nm periodicity of α/β-tubulin heterodimers. Two-color Ex-*d*STORM further reveals the molecular organization of RIM and the synaptic vesicle priming protein Munc13-1 in 44–48 nm ring-like presynaptic structures in neurons. Ex-*d*STORM thus enables nanometer-resolution imaging of endogenous multiprotein complexes in genetically unmodified cells, providing a versatile tool for studying molecular organization in physiological contexts.

## Abstract (web summary)

Eilts and colleagues combine a double-homogenisation expansion microscopy approach with *d*STORM, revealing molecular details of endogenous protein complexes, including microtubules, clathrin lattices and presynaptic vesicle docking sites in cells with nanometre resolution.

## Introduction

Latest single-molecule localization microscopy methods demonstrated superior localization precisions and spatial resolutions in the nanometer range on isolated molecules^1–4^. Similarly, expansion microscopy (ExM) combined with fluorescence intensity fluctuation analysis could resolve the shape and conformation of isolated proteins^5,6^. However, these methods can currently not reliably image protein complexes and arrangements with molecular resolution in cells, respectively. This limitation is mainly caused by inefficient labeling with fluorescent probes, such as IgG antibodies. But also, smaller probes, such as fluorescent proteins and nanobodies, as well as chemical tags, impede stoichiometric labeling of multiprotein complexes and do not permit the labeling densities required to achieve molecular resolution imaging in cells.

A very promising method for high-density labeling of genetically unmodified proteins in cells with minimal linkage error represents post-expansion immunolabeling because dense protein structures are decrowded and epitopes are better accessible by nanometer-sized probes after expansion^7–9^. Particularly, in combination with single-molecule localization microscopy, ExM should thus enable fluorescence imaging of dense protein structures in cells with nanometer spatial resolution. Results reported about single-molecule localization microscopy of expanded samples were motivating but also disclosed problems that must be solved to unleash the full potential of the methodology^10–12^. Among these problems are lower expansion factors and inhomogeneous expansion achieved when trying to preserve protein epitopes for post-expansion immunolabeling^7,13^. Furthermore, the addition of a photoswitching buffer as required for *direct* stochastic optical reconstruction microscopy (*d*STORM) to a swellable polyelectrolyte hydrogel with hydrophilic ionic side groups results in shrinking of the gel^14^. Albeit shrinking can be reduced by re-embedding of charged expanded hydrogels in an uncharged polyacrylamide gel^10,15,16^, the achieved expansion factors were only moderate^10–12^. In addition, carbocyanine dyes, such as Alexa Fluor 647 (AF647), which are the most suitable dyes for *d*STORM, are efficiently destroyed during re-embedding^13,17^. Therefore, sub-10 nm imaging by ExM in cells remains challenging.

In this study, we overcome these previous limitations by using a refined post-expansion immunolabeling and re-embedding protocol that uses two ten-fold robust expansion microscopy (TREx) steps^18^ combined with re-embedding in a neutral hydrogel to achieve 7–8-fold expansion of dense cellular structures and efficient localization of carbocyanine dye-labeled proteins. ExM combined with *d*STORM (Ex-*d*STORM) allowed us to visualize the 8 nm distance between neighboring α-tubulin molecules in microtubules and show the 8-nm periodicity of microtubules by intensity correlation analysis along microtubule filaments^19,20^, and resolve the polyhedral lattice in clathrin-coated pits^21,22^, the molecular organization of the nuclear pore complex (NPC)^23,24^, and the organization of presynaptic proteins of the vesicle docking machinery in genetically unmodified cells^25–27^.

## Results

### Ex-*d*STORM in double-homogenized hydrogels

A broadly applicable nanoscopy method should allow us to resolve the molecular architecture of endogenous proteins and complexes in their physiologically relevant context, i.e., in genetically unperturbed cells, while relying on accessible and widely adopted labeling strategies. Immunolabeling with antibodies best fulfills these criteria, as antibodies are well established, sufficiently sensitive, and available for most target proteins. Unfortunately, the large size of primary/secondary antibodies complexes of ~17.5 nm impedes high-density labeling of cellular structures required to achieve sub-10 nm spatial resolution in cells. However, post-expansion immunolabeling offers the advantage of enhanced epitope accessibility and reduced effective linkage error determined by the expansion factor, thus enabling higher labeling densities and reduced linkage error^10^. On the other hand, since milder homogenization is required to ensure epitope survival for post-expansion labeling with antibodies, the resulting expansion factors are small and often inhomogeneous^7,13,18,28^. This becomes particularly problematic when strong fixation or anchoring methods are used in combination with higher expansion factor protocols. Alternatively, some protocols avoid crosslinking by aldehyde fixation to enable homogenization by denaturation^7,28,29^.

**Fig. 1:**
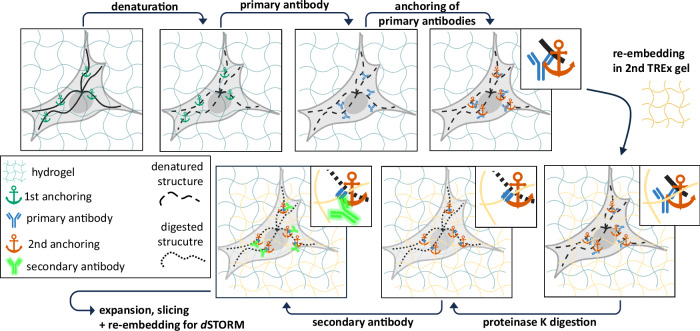
*Double* TREx (*d*TREx) expansion protocol with improved homogenization. Fixed cells are anchored into the hydrogel matrix, followed by a gentle homogenization step through denaturation. This initial, mild treatment allows for efficient labeling of proteins with primary antibodies, which are subsequently incorporated into a second TREx gel. Once the second gel has polymerized, a more rigorous homogenization is carried out using proteinase K digestion. Finally, secondary antibodies are applied to label primary antibody fragments that are anchored into the second gel, and the sample is fully expanded. To prepare the gel for *d*STORM imaging, it is sectioned in thin slices and re-embedded into a neutral gel matrix. Created with Biorender.com. https://BioRender.com/ncu0uin.

To maximize protein retention, epitope survival, and efficient anchoring of proteins into hydrogels, we developed a two-step post-expansion immunolabeling method that uses standard fixation with aldehydes and protein denaturation to preserve epitopes during the initial expansion step. After immunolabeling with primary antibodies, the sample is enzymatically digested by proteinase K treatment followed by immunolabeling with secondary antibodies (Fig. 1). In the first expansion round, cells are fixed with glutaraldehyde (GA) or formaldehyde (FA) and anchored into the hydrogel using GA or a combination of FA and acrylamide (FA + AA). After gelation with TREx monomer solution^18^, proteins anchored in the hydrogel are denatured with sodium dodecyl sulfate (SDS) and dithiothreitol (DTT) at 98 °C to ensure epitope preservation. During immunolabeling with primary antibodies in phosphate-buffered saline (PBS), the hydrogel remains ~3-fold expanded, which increases the distance between adjacent epitopes and promotes higher labeling densities with IgG antibodies. Next, the first hydrogel is crosslinked and re-embedded into a second TREx gel, causing the gel to slightly shrink to a ~2.5-fold expanded state. Anchoring with glutaraldehyde ensures that the primary antibodies are efficiently linked into the second hydrogel matrix. After proteinase K digestion for 45–120 min at 37 °C, the sample is immunostained with fluorophore-labeled secondary antibodies. Finally, the gel is fully expanded (8–9×) and sliced with a razor blade to facilitate diffusion of neutral (non-expanding) gel monomer solution required for stabilization of the expanded sample for *d*STORM imaging (Fig. 1).

We applied the *double* TREx protocol (*d*TREx) on microtubules and clathrin-coated pits in GA-fixed and anchored COS-7 cells and compared the performance with standard TREx post-expansion immunolabeling and *d*TREx, omitting the proteinase K step, i.e., using only denaturation for homogenization. The resulting Airyscan microscopy images clearly showed that TREx, as well as *d*TREx with denaturation alone, result in ruptured expanded microtubules and poorly expanded clathrin-coated pits lacking detailed ultrastructural features about the clathrin coat. In contrast, microtubules expanded by *d*TREx using additional digestion with proteinase K were imaged as continuous filaments (Supplementary Fig. 1). Double homogenization with *d*TREx resulted in expansion factors of the hydrogel (the macroscopic expansion factor) of 7.6 ± 0.3 (s.d.) and 8.4 ± 0.6 (s.d.) using proteinase K digestion at 37 °C for 45 min and 2 h, respectively. Interestingly, *d*TREx using denaturation only showed a similar macroscopic expansion factor of 7.6 ± 0.4 (s.d.) (Supplementary Figs. 1e and 2). On the other hand, the microscopic expansion factors determined from clathrin-coated pits and microtubule diameters showed that double homogenization using *d*TREx results in ~2.5-fold higher expansion (Supplementary Fig. 1f, g), indicating that 8–9-fold expansion of multiprotein complexes requires an additional proteinase K digestion step. These findings underscore the importance of using a balanced protocol that ensures efficient and homogeneous expansion of multiprotein complexes in cells but concurrently preserves protein epitopes for post-expansion immunolabeling.

Having established *d*TREx for homogeneous 8–9-fold expansion of multiprotein complexes in cells, we next focused our efforts on optimizing re-embedding for *d*STORM imaging with carbocyanine dyes, such as AF647. Unfortunately, carbocyanine dyes do not survive the gelation step, most probably due to a radical attack at one of the conjugated double-bonds during polymerization. Primary antibodies labeled with secondary AF647-antibodies only retain less than 10% of their initial brightness after gelation and digestion^13^. Therefore, we tested the use of lower ammonium persulfate (APS) and tetramethylethylenediamine (TEMED) concentrations during re-embedding in the neutral gel to improve signal retention of AF647-labeled secondary antibodies. By titrating the radical starter and catalyst concentration, we observed improved survival of AF647 in ensemble experiments with decreasing APS/TEMED concentration (Supplementary Fig. 3a). Re-embedding of *d*TREx gels with an APS/TEMED concentration of 0.025% resulted in only 10% hydrogel shrinking and ~60% signal retention comparing the fluorescence intensity of identical microtubule filaments in the sample before and after re-embedding (Supplementary Fig. 3b–e). Accordingly, the final expansion factors are 10% smaller. Considering that secondary antibodies are labeled with an average degree of labeling (DOL) of ~3, each antibody should be localized in *d*STORM experiments of expanded samples with high probability using the optimized re-embedding protocol. To avoid chemical destruction of carbocyanine dyes, staining with the secondary antibody can be performed after re-embedding. However, while the measured fluorescence intensities of microtubules labeled either before or after re-embedding with secondary AF647-antibody showed similar fluorescence intensities, the background signal was slightly higher for samples labeled post-re-embedding in the neutral gel (Supplementary Fig. 4). Therefore, we labeled the samples before the re-embedding step in all following experiments.

### Fluorescence intensity correlation analysis along microtubule filaments indicates the 8 nm periodicity of microtubules

To test the performance of *d*TREx expansion and Ex-*d*STORM imaging, we used microtubules as a protein structure that is frequently used as a cellular reference in super-resolution and expansion microscopy^30,31^. Microtubules are polar cytoskeletal polymers that play essential roles in intracellular transport, cell division, and the spatial organization of the cytoplasm. Microtubules are assembled from α,ß-tubulin heterodimers, which stack head-to-tail into polar protofilaments with a periodicity of 8 nm. In most eukaryotic cells, thirteen protofilaments associate laterally to generate a hollow, cylindrical tube with an outer diameter of ~25 nm. This conserved 13-protofilament architecture underlies the mechanical rigidity and dynamic behavior that characterize microtubules^19,32^. The lateral arrangement of protofilaments within the microtubule wall predominantly follows a B-lattice configuration^33^. In this geometry, α-tubulin subunits laterally contact α-tubulin, and β-tubulin contacts β-tubulin in neighboring protofilaments, preserving homotypic lateral interactions across most of the cylinder. However, because the helical pitch of tubulin polymerization does not perfectly match the geometry of a closed 13-protofilament tube, this regular B-lattice arrangement cannot be maintained continuously. As a result, a single discontinuity, termed the seam, forms along the microtubule wall. At this seam, lateral contacts adopt an A-lattice configuration, in which α-tubulin laterally interacts with β-tubulin with an offset of 3 tubulin monomers due to the helical pitch. Thus, canonical 13-protofilament microtubules are characterized by a largely uniform B-lattice organization interrupted by a single A-lattice seam. This structural feature is thought to influence microtubule stability, lattice plasticity, and interactions with microtubule-associated proteins, and represents a defining element of microtubule molecular architecture^19,32–34^.

After immunolabeling, microtubules exhibit an outer diameter of 60 nm, accounting for a linkage error of 17.5 nm defined by the primary and secondary antibody^32^. Although the periodic structure of microtubules is particularly suited as a reference for advanced super-resolution microscopy technologies^1–4^ the limited labeling density along microtubules did so far not allow to directly visualize the 8 nm distance between neighboring tubulin molecules in microtubules and its periodicity. According to information theory, the required density of fluorescent probes must be sufficiently high to satisfy the Nyquist–Shannon sampling theorem^35^. At its most basic level, the theorem states that the mean distance between neighboring localized fluorophores (the sampling interval) must be at least twice as fine as the desired resolution. With 13 protofilaments per ring, 1 µm of microtubule comprises 1625 α-tubulins, of which only a few percent are detected in *d*STORM experiments of unexpanded microtubules labeled for α-tubulin with primary and secondary antibody, which is insufficient to resolve molecular details (Supplementary Fig. 5a). On the other hand, if microtubules are labeled with primary anti-α-tubulin antibodies after the first TREx expansion step, we detected 3–5 times as many localizations in *d*STORM experiments of fully *d*TREx expanded and re-embedded microtubules demonstrating that post-expansion immunolabeling achieves substantially higher labeling densities (Supplementary Fig. 5b).

**Fig. 2:**
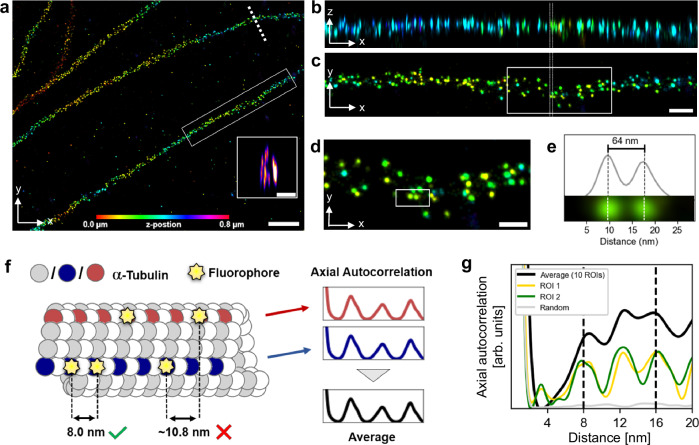
Ex-*d*STORM of microtubules indicates the 8 nm tubulin periodicity. **a** Representative 3D Ex-*d*STORM image of a COS-7 cell using dTREx post-immunostaining for α-tubulin (AF647). The inset shows a microtubule cross-section at the position marked by the dotted line, resolving the hollow, cylinder-like structure of immunolabeled microtubules. **b**, **c** Corresponding xz- and xy-views of the region marked in (**a**). White dashed lines indicate a separation of 64 nm (8 nm unexpanded). **d** Zoomed-in view of the region marked in (**c**). **e** Zoomed-in view of the region marked in (**d**), with the intensity profile superimposed. The two signals are separated by 64 nm (8 nm unexpanded), corresponding to adjacent dimers on the same protofilament. **f** Schematic of a microtubule in B-lattice configuration where α-tubulin was stochastically labeled (yellow). Due to the lateral offset between protofilaments, the 8 nm spacing can only be directly observed along the same protofilament, while axial autocorrelation averaged over all protofilaments reveals the lattice spacing. **g** Autocorrelation function of two individual microtubules (green), and averaged over 10 microtubules (black) (Supplementary Fig. 9). The same number of clusters randomly placed across the filament shows no detectable periodicity (gray) (Supplementary Fig. 8). The intensity profile in (**e**) and the autocorrelation analysis (**g**) were corrected for the final expansion factor of ~8.0 after *d*TREx and re-embedding in a neutral hydrogel. Scale bars (expanded), **a** 2 µm (inset: 200 nm); **b**, **c** 500 nm; **d** 300 nm. Pixel size, 5 nm.

The resulting 3D Ex-*d*STORM images of post-expansion immunolabeled α-tubulin in COS-7 cells indicate the presence of a periodic structure (Fig. 2a–c and Supplementary Fig. 6). However, since microtubules in B-lattice arrangement consist of typically 13 protofilaments with an offset of 0.923 nm between neighboring protofilaments, the periodicity cannot be easily identified by looking at entire stochastically labeled microtubules, where all axial distances occur, not only the 8 nm spacing along a single protofilament (Fig. 2f)^33^. Only when examining the x, y projection of localization clusters detected by 3D *d*STORM along selected short filament areas, 8 nm distances between neighboring clusters can be visualized (Fig. 2b–e and Supplementary Fig. 6). Therefore, to show the 8 nm periodicity, we identified the localization clusters of individual α-tubulins, assigned them to their respective protofilaments and analyzed the distances between them along individual filaments. While the labeling density is not sufficient to see many adjacent α-tubulins, the tubulin periodicity can be observed as peaks in the autocorrelation function of the signal along each protofilament at multiples of the lattice spacing (Fig. 2f, g, Supplementary Figs. 7 and 8).

Here, the autocorrelation function shows peaks at ~4, 8, 12, and 16 nm as expected for microtubules in B-lattice configuration (Fig. 2f, g). The peaks at ~4 and ~12 nm can be explained by the α-tubulin signals along the seam of the B-lattice, considering a single seam of A-lattice contacts^36^, which should give rise to the appearance of a 4 nm displaced, i.e., a 4 and 12 nm periodicity along the seam, respectively. However, the magnitude of the 12 nm peak in the autocorrelation functions is too large to be explained by a single seam in the B-lattice configuration (Fig. 2g). Therefore, we developed a more detailed simulation taking the multiple labeling and expansion steps into account. We assume that the labeling efficiency of the primary antibody is 5%, and it can point in any direction. Enzymatic digestion and ~3× expansion in the second TREx expansion step can then introduce a linkage error of 37.5 nm. Averaging over multiple regions of interests (ROIs) results in the observed axial autocorrelation function with 8 nm periodicity plus a strong additional peak at 12 nm (Supplementary Fig. 7) as observed in our experiments (Fig. 2g). Taking into account experimental uncertainties (localization, antibody orientation etc.), the linkage error of ~37.5 nm (or ~4.7 nm unexpanded) translates into an axial offset of the measured positions close to half the lattice periodicity. Axial distances at multiples of half the lattice periodicity appear as higher harmonics at 32 (4) nm (twice the spatial frequency) in the axial autocorrelation, overlaying the 64 (8) nm periodicity of the tubulin positions.

While the achieved immunolabeling density is still too sparse to reveal a regular and continuous 8 nm lattice, cross-sectional profiles show the hollow space inside the microtubule cylinder (Fig. 2a and Supplementary Fig. 10). Applying this analysis to *d*STORM data of ~3.2× expanded microtubules^10^ fails because of insufficient label density and spatial resolution, which prevents the unequivocal spatial separation of different localization clusters for segmentation into different protofilaments (Supplementary Fig. 11). This result substantiates that an expansion factor of 7–8× (after re-embedding) as provided by *d*TREx using post-expansion immunolabeling in combination with *d*STORM is required to achieve a structural resolution of a few nanometers in genetically unmodified cells.

### Ex-*d*STORM visualizes molecular details of clathrin-coated pits

**Fig. 3:**
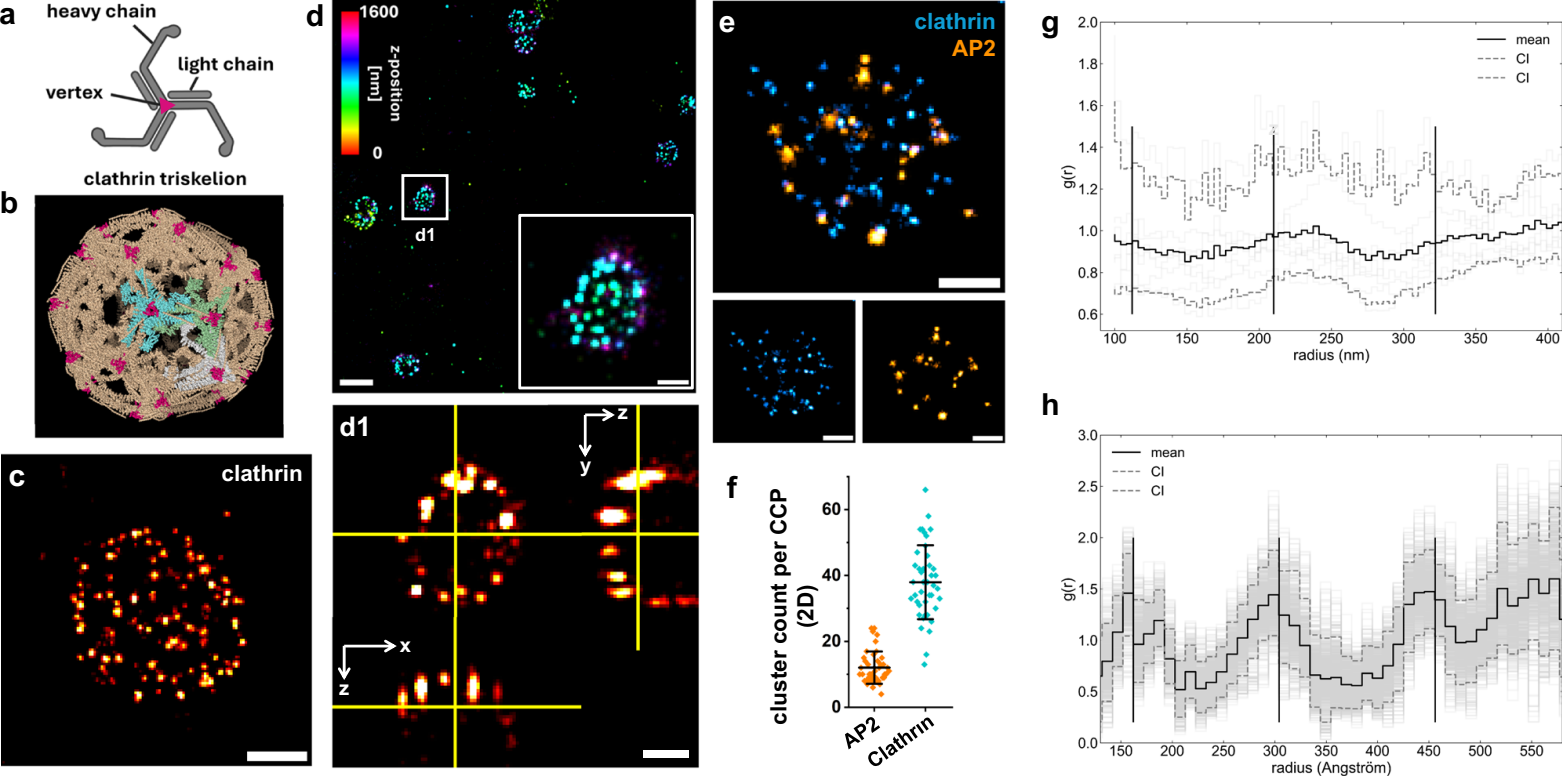
Ex-*d*STORM allows fluorescence imaging of clathrin-coated pits with molecular resolution. **a** Schematic triskelion. **b** Clathrin D6 coat reconstructed in Pymol from PDB structure 1XI4. Three exemplary triskelia are highlighted in cyan, green, and white. Trimerization domains at the vertex of the triskelia are colored pink, representing binding sites for the used antibody. **c** Representative clathrin-coated pit (CCP) visualized by Ex-*d*STORM using the *d*TREx procedure for enhanced homogenization and post-immunostaining for clathrin heavy chain (AF647). Cells were fixed and anchored with GA, and proteinase K digestion was applied for 45 min at 37 °C in the second TREx expansion step. **d** 3D Ex-*d*STORM image. A magnified CCP is shown in the white box and as cross-section (**d1**). **e** 2-color Ex-*d*STORM image of adaptor complex AP2 (AF647) and clathrin (CF568). **f** Localization clusters detected for AP2 and clathrin per CCP using DBSCAN. Scatter dot graph shows single data points, mean values ± sd (AP2 = 12.0 ± 4.9, clathrin = 38.0 ± 11.2). Data from two independent experiments (*n* = 42). **g** Mean radial distribution function for all pairwise localization distances from the recorded *d*STORM localization data in selected regions of interest (*n* = 8). Radial distribution functions are shown relative to those for localizations distributed under complete spatial randomness. Confidence intervals for 5 and 95% are shown (*n* = 40). **h** Mean radial distribution functions for the vertices of the model structure shown in (**b**) (pdb: 1XI4). Radial distribution functions are generated and plotted with 5/95% confidence intervals (*n* = 100 random rotations). Scale bars, **c**, **d1**, **e** 500 nm; **d** 1 µm. Pixel size, **c**, **e** 20 nm; **d**, **d1** 35 nm. Scale bars are given for 7-fold expanded cells. Elements of **a** created in BioRender. https://BioRender.com/ardqsuc.

Another popular reference structure for super-resolution microscopy is clathrin-coated pits^11^. Clathrin-coated pits (CCPs) are curved to spherical, cage-like structures with a size of 50–200 nm located on the plasma membrane responsible for receptor-mediated endocytosis^37,38^. Clathrin-mediated endocytosis involves more than 50 proteins and plays a key role in vesicular trafficking that transports a wide range of cargo molecules from the cell surface to the interior^39^. The clathrin assembly unit is a trimer of three heavy chains and tightly associated clathrin light chains that form a “three-legged structure” termed triskelion with each leg measuring approximately 3 nm in thickness and 52 nm in length, ending in a globular “terminal domain” of 5 nm radius (Fig. 3a)^40^. When assembled into a coat, the legs of the triskelia interdigitate to form ordered lattices with variable ratios of predominantly pentagonal and hexagonal faces (Fig. 3b)^21^.

For visualization of CCPs by Ex-*d*STORM, we immunolabeled the C-terminal region of clathrin heavy chain in GA fixed and anchored COS-7 cells. The C-terminal region of the clathrin heavy chain is located close to the vertex of the triskelion^41^. After implementing the *d*TREx procedure with additional proteinase K digestion for 45 min at 37 °C, gels were labeled with secondary antibodies and re-embedded in the neutral hydrogel. Ex-*d*STORM images clearly resolve the polyhedral lattice of expanded CCPs with an average size of 1 µm (Fig. 3c and Supplementary Fig. 12). The vertices of CCPs are visualized by clusters of localizations resulting from repetitive blinking of AF647- labeled secondary antibodies. 3D-Ex-*d*STORM images show the curvature of a just being formed CCP at the plasma membrane (Fig. 3d and Supplementary Fig. 13). Clathrin shells with average diameters of ~80 nm contain about 35–40 triskelia and 20–25 heterotetrameric AP2 adaptors. AP2 adaptors link the clathrin coat and the membrane bilayer, and they are the principal cargo-recognition molecules^41^. In our Ex-*d*STORM images, we counted on average 12 AP2 and 38 triskelia, assuming that each localization cluster detected marks the vertex of a triskelion (Fig. 3e, f). This result confirms that our optimized *d*TREx and re-embedding protocol enables high-density immunolabeling of endogenous proteins in cells and ensures efficient survival and localization of AF647-labeled antibodies.

Next, we determined the pairwise distances between all localizations from 2D-projected Ex-*d*STORM images of clathrin. The resulting radial distance distribution from all individual localizations of individual CCPs revealed peak positions around 100–150, 200–250, and 350–400 nm corresponding to distances of 14–21, 29–36, and 50–57 nm, respectively, assuming 7.0-fold expanded CCPs after re-embedding (Fig. 3g and Supplementary Fig. 14). The experimentally detected distribution is in good agreement with that for 2D-projected antibody epitope positions generated from the published D6 clathrin coat (pdb: 1XI4) which revealed peak distances of ~16, ~30, and ~46 nm (Fig. 3h and Supplementary Fig. 15). The peaks match the distance between adjacent vertices of the clathrin lattice, which have been measured by electron microscopy to 18–20 nm^41,42^ and 31–35 nm to the second-nearest vertex. The radial distance distribution of individual CCPs indicates some variation in peak positions and overall distribution, possibly resulting from *d*STORM blinking effects and an estimated up to 10% expansion factor variation between different experiments. With an expansion factor of 7× after re-embedding, we determined an effective localization precision of ~2.5 nm in lateral and 9 nm in axial direction in our CCP Ex-*d*STORM experiments, even at an imaging depth of up to 100 µm. Here, the effective localization precision refers to the predicted localization precision from *d*STORM experiments divided by the expansion factor. The values demonstrate that *d*TREx with standard immunolabeling in combination with Ex-*d*STORM achieves a spatial resolution which is comparable to the size of a typical protein molecule in cells.

To further demonstrate the performance of the method for resolving molecular details of cellular multiprotein complexes, we used the NPC as a well-characterized structural benchmark. NPCs rank among the largest macromolecular assemblies in cells and are composed of approximately 30 distinct proteins, termed nucleoporins (NUPs), which are present in multiple copies and arranged in an 8-fold rotational symmetry^23,24,43^. While most super-resolution microscopy investigations of NPCs have been performed with genetically modified NUPs to improve the labeling efficiency^2,3,44,45^, we used COS7 cells and post-expansion immunolabeling to visualize endogenous NUP96 by Ex-*d*STORM. The NUP96 signals showed a mean peak-to-peak distance of 893 ± 48 nm (s.d.) translating into a microscopic expansion factor of 8.3× using the mean diameter of NPCs marked by NUP96 of 107 nm (Supplementary Fig. 16a–c)^45^. NUP96 is present in 32 copies per NPC and contributes to both the cytoplasmic and nucleoplasmic ring, with 16 copies in each ring. Within each ring, NUP96 is organized into eight dimers arranged at regular 12 nm intervals^24,45^. Ex-*d*STORM allowed us to resolve endogenous NUP96 dimers in the nuclear envelope separated by 11.9 ± 1.8 nm (mean ± s.d.) using standard immunolabeling (Supplementary Fig. 16d–g).

### The presynaptic docking machinery is arranged in ring-like structures of different stoichiometry

Having established an efficient method for sub-10 nm fluorescence imaging in cells, we reasoned that *d*TREx and Ex-*d*STORM can be used advantageously to resolve the molecular organization of Munc13-1 and Rab3-interacting molecule (RIM), two proteins that are important components of the vesicle fusion machinery in neurons at the presynaptic membrane^46–48^. In central synapses, the readily releasable pool typically consists of 5–10 synaptic vesicles (SVs) docked within 5 nm of the presynaptic plasma membrane^25^. The release of neurotransmitters from SVs is remarkably fast, requiring cooperation of multiple SNARE (soluble N-ethylmaleimide-sensitive-factor attachment receptor) complexes—a key element of the process that mediates contact between SV and plasma membrane—to achieve this feat^25,46,47^. Using cryo-electron tomography, it has been shown that efficient binding of single SV requires a minimum of 6 copies of the chaperone Munc13-1 to initiate SV priming^25,49^. Accordingly, 6 copies of Munc13-1 can assemble into a closed hexagon with a diameter of ~30 nm, which could, in cooperation with other presynaptic proteins of the docking machinery, form the template for SV capturing, priming, and release. Depending on the state of vesicle docking, the orientation of Munc13-1 on the membrane changes and therefore the size of the hexagon might be variable^25,50^.

The scaffolding protein RIM is closely interacting with Munc13-1 at presynaptic SV docking sites by binding via its Zinc-finger domain to the Munc13-1 C2A domain, forming RIM-Munc13-1 heterodimers^51^. Using immunolabeling and *d*STORM, it was shown that both RIM and Munc13-1 form discrete nanoclusters at the presynapse that could represent the postulated template for SV docking^52,53^. Achieving an improved immunolabeling efficiency and a 7–8-fold higher spatial resolution, we hypothesized that our *d*TREx protocol in combination with Ex-*d*STORM can resolve these discrete nanoclusters and visualize the molecular organization of SV docking sites at the presynaptic membrane. Therefore, we immunolabeled Munc13-1 with AF647 and RIM1/2 (further referred to as RIM) with CF568 in hippocampal mouse neurons before re-embedding of *d*TREx-expanded hydrogels. The expansion factor was determined to ~7.5 after re-embedding by comparing neurons immunolabeled for neurofilament-H before and after *d*TREx (Supplementary Fig. 17).

**Fig. 4:**
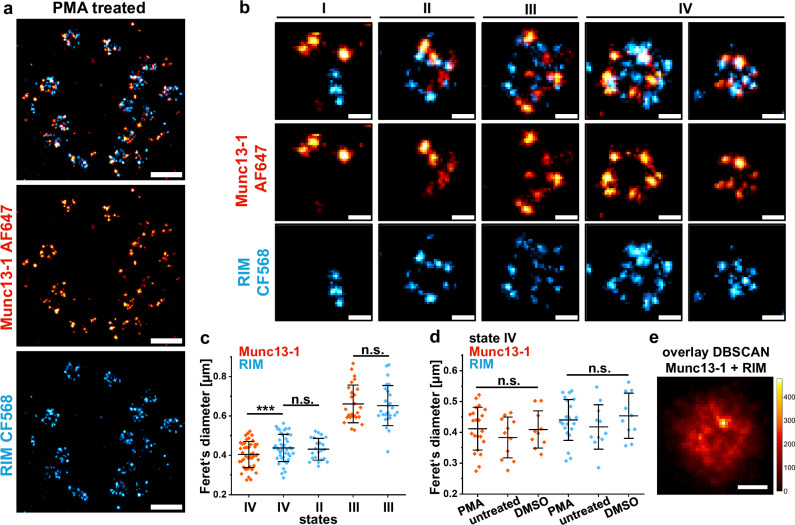
*d*TREx with two-color Ex-*d*STORM reveals the organization of Munc13-1 and RIM in hippocampal mouse neurons. **a** Two-color Ex-*d*STORM images showing a frontal view of a ~7.5-fold expanded active zone in a hippocampal neuron. The sample was treated with 2 µM phorbol 12-myristate 13-acetate (PMA) for 30 min before fixation to increase the probability of detecting active vesicle fusion sites^54^. RIM (CF568, blue) and Munc13-1 (AF647, red) are organized in ring-like structures of different degrees that might represent SV docking sites. **b** Magnified Ex-*d*STORM images of individual Munc13-1 and RIM arrangements in active zones show regions of varying sub-structures from different synapses categorized in four different states. **I** Munc13-1 and RIM unorganized. **II** Only RIM shows ring-like arrangements. **III** Munc13-1 and RIM are organized in substructures with a diameter >500 nm. **IV** Munc13-1 and RIM are both organized in ring-like structures with varying diameters <500 nm. **c** Comparison of substructures, exemplary shown in (**b**) and Supplementary Fig. 19 of all three conditions (PMA, untreated control, DMSO control). Feret’s diameter of structures determined by the polygon tool (Supplementary Fig. 21). In state IV, ring-like structures of RIM exhibit a larger diameter (437 ± 69 nm) than Munc13-1 (404 ± 66 nm; *n* = 43), corresponding to ~48 nm (RIM) and ~44 nm (Munc13-1) diameters of unexpanded structures. The RIM diameter in state II (RIM = 431 ± 55 nm; *n* = 22) is identical to state IV. Munc13-1 and RIM diameters in state III are similar, with 653 ± 102 nm (RIM) and 661 ± 96 nm (Munc13-1; *n* = 26) corresponding to diameters of ~83–84 nm of unexpanded structures. Data were obtained from three independent experiments and neuronal cultures for each condition. Scatter dot graphs show single data points, mean ± s.d. *P*-values of two-sided paired sample t-test (for Munc13-1 versus RIM in state IV, *p* = 0.0002; Munc13-1 versus RIM in state III, *p* = 0.75) and two-sided unpaired t-test (for RIM state II versus state IV, *p* = 0.70) shown as *p* < 0.001 (***) and *p* > 0.5 (n.s., non-significant). **d** Munc13-1 and RIM diameters of ring-like structures determined in state IV (from **c**) under different experimental conditions (PMA treated, untreated control and DMSO treated control) show no significant (n.s.) differences (one-way ANOVA with post-hoc Tukey test). Scatter dot graphs show single data points, mean  ± s.d. **e** Overlay of localization clusters (*n* = 73) for Munc13-1 and RIM signals. Clusters were determined by DBSCAN and selected based on convex hull area and circularity, shifted by their centroid position and re-binned as overlay figure. The spatial distribution indicates the variety of substructures hiding the center hole that clearly appears in selected clusters. The color scale represents localizations per pixel. Scale bars, **a** 1 µm; **b**, **e** 200 nm. Scale bars show ~7.5× expanded dimensions after re-embedding in the neutral hydrogel.

Since phorbol esters have been reported to increase neurotransmitter release by activating Munc13-1, hippocampal neurons were treated with 2 µM phorbol 12-myristate 13-acetate (PMA) for 30 min before fixation to increase the probability of detecting active vesicle fusion sites^54^. In the acquired 2-color Ex-*d*STORM images we focused on investigating frontal views of synapses to determine the molecular organization of Munc13-1 and RIM at docking sites at the presynaptic membrane (Fig. 4a and Supplementary Fig. 18). Indeed, using post-expansion immunolabeling by *d*TREx in combination with two-color Ex-*d*STORM visualizes the organization of Munc13-1 and RIM in ring-like structures at active zones in hippocampal neurons under different conditions with an effective localization precision of ~2.5 nm (Fig. 4a and Supplementary Fig. 19). To test for spatial clustering, we computed Ripley’s h-function which confirmed the visual impression of clustering on various length scales. The h-function computed for simulated data with homogeneously distributed emitters, which only exhibit *d*STORM-based repetitive blinking, remains outside the 5–95% confidence interval of h-functions for all experimental datasets up to ~100 nm (corresponding to ~ 750 nm in the *d*TREx expanded hydrogel) (Supplementary Fig. 20).

Visually, two-color Ex-*d*STORM images indicate that Munc13-1 and RIM are organized in a variety of assemblies in active zones, most of them showing ring-like structures. To distinguish the different structures and classify them into four different states we used the following criteria (Fig. 4a, b, Supplementary Figs. 18 and 19): unorganized appearance of both signals (state I), only RIM exhibits ring-like arrangements (state II), Munc13-1 and RIM signals are organized in arrangements with diameters >500 nm (state III), and both, Munc13-1 and RIM are organized in ring-like structures with varying diameter <500 nm (state IV). While state I might represent Munc13-1 and RIM molecules that are in the process of forming SV docking sites, state II arrangements indicate that RIM forms ring-like structures in active zones more efficiently. Possibly preformed or faster assembled RIM rings recruit Munc13-1 to SV docking sites. State III might represent overlapping SV docking sites that cannot be clearly resolved under the applied experimental conditions. Finally, state IV shows smaller rings with diameters of ~300–500 nm, probably representing established SV docking sites with varying stoichiometry of Munc13-1 and RIM (Fig. 4a–c and Supplementary Figs. 18 and 19). Here, the measured diameters of RIM structures were significantly larger than those of Munc13-1 structures (Fig. 4d).

Considering the expansion factor of 7.5× and the size of primary and secondary antibodies, we determined average ring diameters for state IV structures in active zones of hippocampal neurons of ~44 nm for Munc13-1 in accordance with previous reports^25,55^ and ~48 nm for RIM. Here, it has to be considered that the measured ring diameters might be overestimated because we are labeling the N-terminus of Munc13-1 and RIM. Munc13-1 is a large protein with a size of ~20 nm, whereby the immunolabeled N-terminus directs away from the SV docking site and the C-terminus is interacting with the synaptic vesicle^56^. The sizes of the larger Munc13-1 and RIM clusters in state III did not show a significant difference (Fig. 4c). In addition, we did not detect significant differences between the sizes of ring-like structures in state IV measured under different experimental conditions PMA treated, untreated and DMSO (control) (Fig. 4d). Visual analysis of ring-like state IV structures under different experimental conditions indicates that we detect more ring-like assemblies after phorbol ester treatment which supports the idea that state IV represents functional SV docking sites (Supplementary Fig. 19).

Finally, we analyzed the number and size distribution of the ring-like structures by detecting localization clusters in the combined Munc13-1 and RIM signals with a DBSCAN algorithm and selecting cluster subsets based on convex hull area and circularity (Supplementary Figs. 22–25)^57,58^. It must be noted that this procedure is not capable of identifying all ring-like structures because there is too much overlap between individual localization clusters. However, the cluster selection procedure selects many well-isolated clusters in an objective and reproducible way to compare cluster formation under different conditions. The overlay image of this cluster selection (Fig. 4e and Supplementary Fig. 23) and the calculated radial distribution function for signal clusters indicate identical length scales for Munc13-1 and RIM in and between all experimental conditions (Supplementary Fig. 24).

Interestingly, applying only denaturation for homogenization (using a standard TREx gel)^18^ does not lead to sufficient expansion of SV docking sites in active zones, highlighting the importance of the optimized *d*TREx protocol (Supplementary Fig. 26). Overall, our results demonstrate that active zones in hippocampal neurons exhibit strong differences in local protein density. In addition, SV docking sites show a large variation in composition and diameter, giving rise to the observation of different docking site arrangements.

## Discussion

Here, we introduce *d*TREx that enables a ~4-fold higher labeling density of microtubules by post-expansion immunolabeling with primary and secondary antibodies and thus allows, in combination with Ex-*d*STORM, to resolve the 8 nm distance between neighboring α-tubulin molecules and fine details of the molecular architecture of clathrin-coated pits. *d*TREx excels as it allows efficient expansion (8–9× before and 7–8× after re-embedding in the neutral hydrogel) combined with improved immunolabeling efficiency and reduced linkage error. Optimization of the re-embedding conditions ensures that the majority of cyanine dyes (AF647) survive re-embedding and thus allow *d*STORM imaging in expanded gels. Using spontaneously blinking dyes avoids re-embedding, but the results obtained so far are less promising^10,59^.

Our results clearly show that the ability to resolve complex multiprotein structures in cells is not as much an issue of the localization precision provided by the super-resolution microscopy technique^1–4^ as it is of the labeling density. The achieved effective localization precision of a few nanometers in combination with a ~4-fold higher immunolabeling density enables researchers to uncover the nanoarchitecture of genetically unmodified cells using established immunolabeling and fluorescence imaging methods with so far unmatched structural resolution. The availability of imaging technologies that can visualize the nanoscopic organization of endogenous proteins in cells is important because transiently or constitutively overexpressed proteins can cause a multitude of problems, especially in super-resolution microscopy experiments, including protein aggregation and aberrant organelle morphology and generate misleading nanoscale structures and clustering artifacts^60–62^. Our approach is broadly applicable since antibodies are available for efficient immunolabeling of most cellular proteins. However, the primary antibody must be well-suited to efficiently bind to epitopes of denatured proteins. For proteins that tend to aggregate during heat denaturation, this step may have to be adjusted using, e.g., a lower denaturation temperature.

Our results demonstrate that Ex-*d*STORM of *d*TREx expanded hippocampal neurons is ideally suited to unravel the molecular organization of synaptic proteins in crowded compartments. It allowed us to resolve the organization of Munc13-1 and RIM in active zones in ring-like assemblies of different diameters and geometries. The observation of a ring-like organization of RIM in active zones is supported by recent models that suggest that RIM is initially responsible for tethering of SVs to prepare them for membrane fusion^63,64^. Different arrangements of the SV docking machinery in active zones might be well explained by the different states of vesicle docking, priming, and exocytosis^25^. The diameters of the ring-like structures observed for Munc13-1 and RIM depend accordingly on the respective state of the individual docking site. In addition, absolute diameters of these ring-like structures are difficult to determine given the uncertainties in linkage errors estimated for IgG antibodies after expansion and local variations in expansion factors^9–13,65^. Nevertheless, the estimated ring diameters of ~44 nm for Munc13-1 and ~48 nm for RIM are in consideration of the antibody epitope comparable to protein density rings of ~38 nm observed under primed vesicles by cryoelectron tomography^55^. Altogether, our results demonstrate that our optimized *d*TREx protocol in combination with Ex-*d*STORM provides a versatile method to resolve so far unresolvable molecular details of the molecular organization of cells.

## Methods

We confirm that our research complies with all relevant ethical regulations. C57BL/6J mice were maintained on a 12 h light/12 h dark cycle at a temperature of 22–24 °C, relative humidity of 50–60% and access to food and water ad libitum. Experiments complying with all ethical regulations were approved by the responsible governmental authorities (“Regierung von Unterfranken”) and conducted in accordance with the German Animal Welfare Act (TierSchG).

### Reagents

B-27™ Plus Supplement (no. A3582801), formaldehyde methanol-free (no. 28906), GlutaMAX™ (no. 35050038), 10× HBSS (no. 14060040), Neurobasal™ Plus (no. A3582901), proteinase K (no. AM2548), sodium dodecyl sulfate (SDS, AM9820), Triton™ X-100 (Triton, no. 28314), trypsin-EDTA (no. 25300054), and Tween 20 (no. 28320) were purchased from Thermo Fisher Scientific. Acetic acid (no. A6283), acrylamide (AA, no. A4058), ammonium persulfate (APS, no. A7460), (3-aminopropyl)triethoxysilane (APTES, no. 440140), bovine serum albumin (BSA, no. A7030), cysteamine hydrochloride (MEA, no. M6250), dithiothreitol (DTT, no. 646563), DMEM/F12 medium (no. D8062), ethanol (no. 32205), ethylenediaminetetraacetic acid disodium salt dihydrate (EDTA, no. E1644), ethylene glycol-bis(2-aminoethylether)-N,N,N′,N′-tetraacetic acid (EGTA, no. 03777), fetal bovine serum (FBS, no. F7524), formaldehyde (FA, no. F8775), gentamicin (no. G1272), glucose (no. G7528), glucose oxidase (no. G2133), glutaraldehyde (GA, no. G5882), guanidine hydrochloride (no. 50933), HEPES (no. H0887), KOH (no. P1767), 2-(N-morpholino)ethanesulfonic acid (MES, no. M3671), MgCl_2_ (no. 442615), NaCl (no. S7653), N,N′-methylenebisacrylamide (Bis, M1533), PBS (no. D8537), penicillin-streptomycin (no. P4333), poly-D-lysine (PDL, no. P6407) and N,N,N′,N′-tetramethylethylenediamine (no. T7024) were purchased from Merck. Catalase (no. 6025.1) was purchased from Carl Roth GmbH. Phorbol 12-myristate 13-acetate (PMA, Cay10008014-1) was purchased from Biomol GmbH.

### Antibodies

Rabbit anti α-tubulin (Abcam, no. ab18251), mouse anti α-tubulin (Merck, T6199), rabbit anti clathrin heavy chain (Abcam, no. ab21679) and mouse anti AP2 (Proteintech, no. 68349-1-Ig) were used at a concentration of 10 µg/ml. The working concentrations for neuron antibodies, rabbit anti- Munc13-1 (Synaptic Systems, no. 126103) and guinea pig anti RIM1/2 (Synaptic Systems, no. 140 205) were 15 µg/ml. Chicken anti-neurofilament-H (Biolegend, no. 822601) was applied at 19 µg/ml for pre-expansion staining and at 38 µg/ml for post-expansion staining. Rabbit anti-NUP98-96 (Proteintech, no. 12329-1-AP) was applied at a concentration of 15 µg/ml. Secondary antibodies donkey anti rabbit AF647 (ThermoFisher, no. A-31573), donkey anti mouse AF647 (ThermoFisher, no. A31571), donkey anti guinea pig CF568 (Merck, no. SAB4600469) and goat anti rabbit CF568 (Merck, no. SAB4600085) were used at a concentration of 20 µg/ml. Donkey anti chicken (Jackson Immunoresearch, no. 703-005-155) was conjugated with ~7-molar excess of N-hydroxysuccinimidyl-ester-CF568 (Merck, no. SCJ4600027) using Zeba™ Spin Desalting Columns (Fisher-Scientific, no. 87766) according to the instruction manual. The conjugated antibody was applied at a concentration of 10 µg/ml (pre-expansion) and 20 µg/ml (post-expansion).

### Cell culture

COS-7 cells (CLS Cell Line Service GmbH) were cultured at 37 °C with 5% CO_2_ in DMEM/F12 medium supplemented with 10% FBS and 1% penicillin-streptomycin. ~50,000 cells were seeded on 12 mm round cover glasses and grown for ~24 h until fixation.

### Preparation of primary hippocampal mouse neurons

Neurons were isolated from E18 C57BL/6J mice from both sexes, in accordance with approval from the Bavarian state authorities (Government of Lower Franconia) and in accordance with the European guidelines for the care and use of laboratory animals. Hippocampal tissue was enzymatically digested using 0.05% trypsin-EDTA at 37 °C for 15 min, followed by two rinses in HBSS. The HBSS solution consisted of 10× HBSS, 250 μl gentamicin, 3.5 ml of 1 M HEPES, and was diluted to a total of 500 ml using distilled water. After digestion, the tissue was gently dissociated using pipettes with different pore sizes in a Neurobasal culture medium. This medium was composed of 100 ml Neurobasal™ Plus, 1 ml GlutaMAX™, 50 μl gentamicin, and 2% B-27™ Plus Supplement. The dissociated neurons were seeded onto 12 mm coverslips that had been coated with PDL (0.1 mg/ml, incubated at room temperature for 1 h and rinsed twice with distilled water). Each coverslip received 40,000 cells, which were then cultured in Neurobasal medium at 37 °C in a 5% CO_2_ incubator for 21 days. Half of the medium was replaced once per week. Optionally, neurons were treated with 2 µM PMA or 0.2% DMSO (solvent for PMA) for 30 min at 37 °C immediately before fixation. To fix the neurons, they were treated with 4% methanol-free formaldehyde for 15 min at room temperature and washed 3 times with PBS.

### *d*TREx with double homogenization

COS-7 cells were fixed in two steps with cytoskeleton buffer (CB-buffer, containing 10 mM MES, 150 mM NaCl, 5 mM EGTA, 5 mM glucose and 5 mM MgCl_2_, pH 6.1). First, cells were incubated with 0.3% GA + 0.25% Triton in CB buffer for 60 s at 37 °C and immediately afterward fixed with 2% GA in CB buffer for 10 min. For NPC visualization, COS-7 cells were fixed first with 0.3% GA and 0.25% Triton for 60 s at 37 °C and then directly with 2.4% FA for 20 min. Primary hippocampal neurons (DIV21) were fixed with 4% methanol-free formaldehyde (FA) for 15 min. After three washing steps, COS7-cells were crosslinked with 0.25% GA for 15 min, and neurons with a 4% FA + 30% AA overnight at 37 °C^29^. Samples were washed with PBS twice and dipped in TREx monomer solution once before gelation^18^. TREx monomer solution contained 1.1 M sodium acrylate, 2 M AA, 0.009% Bis, 1× PBS, 0.15% TEMED and 0.15% APS. Coverslips were then flipped on a 60 µl drop of TREx monomer solution with the cell side down. Polymerization was performed on ice for 15 min and then at RT for 1.5 h. Gels were then homogenized in pre-heated denaturation buffer consisting of 200 mM SDS, 200 mM NaCl and 50 mM Tris (pH 8) for 1 h at 98 °C. Right before this step, 65 mM DTT was added to the buffer. The denaturation buffer was then removed thoroughly by washing 5× with ~5 ml prewarmed (37 °C) PBS on a rotating wheel. Primary antibodies were incubated at a concentration of 10–15 µg/ml in 5% BSA iteratively overnight at 4 °C and again with fresh antibody solution for 3 h at 37 °C. Unbound antibodies were removed by washing 3 × 15 min with 0.1% Tween 20 in PBS (PBST) on a rotating wheel. To enable an additional digestion step, primary antibodies were anchored into a second TREx gel. Therefore, the partly expanded samples (~3 fold) were crosslinked with 0.25% GA for 20 min and washed three times for 15 min with PBS. For re-embedding into a second TREx gel, samples were incubated twice for 30 min in 500 µl TREx monomer solution containing 0.03% TEMED/APS. This caused the gels to shrink to a ~2.5-fold expanded state. The monomer solution was removed, and the gel was placed between two coverslips and incubated for 1.5 h at 37 °C in a N_2_-filled humidified chamber. For more efficient homogenization - additive to denaturation - a digestion step was included. Therefore, gels were incubated with 8 U/ml proteinase K in digestion buffer (50 mM Tris + 1 mM EDTA + 0.8 M guanidine HCl + 0.5% Triton) for 45 min (CCPs, neuronal proteins and NPCs) or 2 h (microtubules) at 37 °C. Gels were then thoroughly washed 5× for 15 min with cold 0.1% PBST. Secondary antibodies were incubated at a concentration of 20 µg/ml in 5% BSA overnight and again with fresh antibody solution for 3 h at 37 °C. After 3 × 15 min washing steps with 0.1% PBST, gels were fully expanded in ddH_2_O, preferably overnight, before imaging or re-embedding.

### Post-labeling procedure with denaturation only

Gels were processed in the same way as described in the previous section (*d*TREx with double homogenization) until after the primary antibody incubation step. After washing unbound primary antibodies, secondary antibodies were incubated at a concentration of 20 µg/ml in 5% BSA overnight at RT or 3 h at 37 °C. Following 3 × 15 min washing steps with 0.1% PBST, gels were fully expanded in ddH_2_O preferably overnight before imaging or re-embedding.

### Silanization of coverslips

Gels were placed on silanized 24-mm round coverslips (thickness 1.5H, Carl Roth, PK26.2) for imaging and re-embedding for *d*STORM. Coverslips were previously cleaned via three 15 min washing steps with first ddH_2_O, then 1 M KOH and in the end 99% ethanol in an ultrasound bath. After drying, coverslips were incubated with silane solution consisting of 0.001% APTES, 80% ethanol and 0.02% acetic acid in ddH_2_O. Each coverslip was covered with ~250 µl of silane solution and left to completely evaporate under a fume hood. Coverslips were washed twice with 99% ethanol, left to dry and stored at −20 °C.

### Re-embedding for *d*STORM

To prevent shrinking of expanded gels in salt-containing imaging buffer, gels were re-embedded into a neutral gel. Therefore, we followed the previously published re-embedding method with some modifications^10^. All incubation steps were conducted in 2 ml tubes on a spinning wheel. First, the expanded gels were cut with a razor blade into thin (~0.5–1 mm) slices to facilitate diffusion of buffer into the gels. A crosslinking step with 0.25% GA in ddH_2_O for 20 min was implemented, followed by 3 × 15 min washing steps with ddH_2_O. Gels were incubated in re-embedding solution (10% AA + 0.15% Bis in ddH_2_O) containing 0.025% TEMED and 0.025% APS twice for 30 min. After removing the solution, gels were placed on silanized 24 mm coverslips with the cell side down. Another coverslip was placed on top. Polymerization occurred for 1.5 h in a N_2_-filled humidified chamber at 37–40 °C.

### Airyscan imaging

Airyscan images were captured using an LSM 900 microscope equipped with Airyscan 2 (Zeiss) in super-resolution (SR) imaging mode, utilizing a C-Apochromat 40×/1.2 numerical aperture (NA) water-immersion objective (Zeiss) for expanded gels. For expansion factor determination, a Plan-Apochromat 63×/1.4 NA oil objective (Zeiss) was used for pre-expansion recordings and a Plan-Apochromat 20×/0.8 NA or Plan-Apochromat 10×/0.45 NA air objective for post-expansion recordings. Appropriate excitation wavelengths and filter settings for the dyes were chosen through the dye presets in ZEN blue software (Zeiss, version 3.5). To compare the fluorescence intensity of AF647 before and after re-embedding, all acquisition parameters were kept the same. All images were processed using the standard strength mode for 3D Airyscan processing.

### *d*STORM image acquisition

Re-embedded gels were incubated in switching buffer for 45 min and immersed in fresh switching buffer immediately before imaging. Switching buffer consisted of 100 mM cysteamine hydrochloride (MEA) and oxygen scavenger system (5% glucose, 11 U/ml glucose oxidase and 220 U/ml catalase) in 1× PBS (pH adjusted to pH 7.6). Imaging chambers were purged with Argon gas and sealed with parafilm. *d*STORM measurements were performed on an inverted fluorescence widefield microscope (Olympus, IX-71) equipped with a nosepiece stage (Olympus, IX2-NPS) and two EMCCD cameras (Andor iXon Ultra DU-897). Biplane 3D images were acquired with a 60× oil objective (Olympus, NA 1.45 PlanApo) and HILO (highly inclined and laminated optical sheet) illumination using 4–7 kW/cm^2^ of an appropriate laser (Toptica, iBeamSmart 640-S_11598). For recording on two EMCCD cameras simultaneously, a two-channel image splitter (TwinCam, Cairn Research) equipped with a 50/50 beamsplitter (Cairn Research) was used, and the cameras were synchronized by a pulse generator (DG535, Stanford Research Systems). Two identical bandpass filters (RazorEdge LP Edge Filter 647 RU, Semrock) were positioned in front of the two cameras. A dichroic mirror (FF410/504/582/669, Chroma) was placed in front of the objective to separate excitation and emission light. In addition, a quarter-wave plate (Thorlabs, SAQWP05M) was mounted for excitation with circular polarized light. Subsequently, 45000-60000 frames were acquired on both cameras with a frame rate of 50 Hz. A UV-laser pulse was added if required to return more fluorophores to the on-state. For calibration, 100 nm microspheres (Invitrogen, T7279) were prepared by incubating ~10^5^ beads/ml in 1×PBS (Sigma-Aldrich, D8537-500ML) with 50 mM MgCl_2_ (Sigma-Aldrich, M9272-500G) adjusted to pH 7.4, for 15 min using eight chambered cover glass systems with high-performance cover glass (Cellvis, C8-1.5H-N), followed by three washing steps prior calibration experiments. The calibration measurements were performed by using a piezo scanner (Pifoc, Physik Instrumente) driven with a LVPZT servo controller (E-662, Physik Instrumente) to move the objective.

For two-color *d*STORM imaging, an oil-immersion objective (APON 60×, numerical aperture 1.49; Olympus) was employed. Alexa Fluor 647 and CF568 were excited by 5–6 kW/cm^2^ of a 639 nm laser and 4–7 kW/cm^2^ of a 561 nm laser (Genesis MX 639-1000 STM and Genesis MX 561-500 STM, Coherent). A dichroic mirror (FF410/504/582/669, Chroma) was used to separate excitation from emission light. A beamsplitter (630 DCXR customized, Chroma) projected emission light through two different bandpass filters (607/70 and 679/41 BrightLine series, Semrock) on two separate EMCCD cameras. Twenty thousand to thirty thousand frames of the two channels were acquired sequentially in HILO illumination with a frame rate of 50 Hz. Images were reconstructed in the open-source software rapidSTORM 3.3^66^, usually with a pixel size of 20 nm. For two-color alignment, 0.2 µm fluorescent microspheres (Invitrogen™, T14792) were imaged in each channel, and an alignment matrix was created with the ImageJ plugin bUnwarpJ. The matrix was then applied to the reconstructed images.

### 3D biplane analysis

For extracting 3D information of biplane *d*STORM recordings, an intensity-based analysis routine was used. First, the measured image stacks were analyzed with rapidSTORM 3.3^66^. Therefore, the FWHM was set to 360 nm, the intensity threshold was set to 250 ADC and the fit window radius to 1100 nm. The corresponding localization files from both cameras were then analyzed further using a custom-written Python script to calculate the 3D intensity ratios. The calibration file, as well as the sample files measured, were analyzed in the same way as described elsewhere in detail, but focused on the rapidSTORM analyzed intensity values for calculating the intensity ratios^67,68^. In brief, calibration curves were calculated from localizations detected in both channels by nearest neighbor assignment; localizations separated by more than 300 nm after channel transformation were discarded. For each paired localization, the intensity ratio ${I}_{{{\mathrm{1,2}}}}=\frac{{I}_{1}-{I}_{2}}{{I}_{1}+{I}_{2}}$ was calculated based on the measured spot intensities, where ${I}_{1}$ and ${I}_{2}$ represent the intensities in channel 1 and channel 2, respectively. Final z-coordinates were obtained from calibration lookup tables and corrected for refractive index mismatch using a scaling factor of 0.71 (assuming refractive indices of buffer and glass, numerical aperture NA = 1.45, emission wavelength λ_em_ = 668 nm and isotropic emission). For visualizing 3D images, ImageJ (version 2.16.0/1.54g) and ThunderSTORM (version 1.3) were used.

### Ensemble measurement of AF647 survival under different gelation conditions

Fluorophore stability under gelation-like conditions was assessed by monitoring time-dependent fluorescence intensity of Alexa Fluor 647 conjugated antibodies in solutions containing varying concentrations of APS and TEMED. Measurements were performed in quartz glass cuvettes using a Jasco FP-8350 spectrofluorometer. Excitation was set to the absorption maximum of Alexa Fluor 647, and emission intensity was recorded every 30 s. The temperature of the measurement chamber was adjusted to 37 °C. Photobleaching was minimized by opening the excitation shutter only during acquisition intervals. For measurements, a donkey anti-rabbit IgG conjugated with AF647 (ThermoFisher, A31573; degree of labeling ~5) was diluted to 1 µM in 1× PBS. APS and TEMED were then added to the antibody solution at final concentrations corresponding to three gelation protocols: TREx (0.15%), re-embedding (0.05%), and modified re-embedding (0.025%). Measurements were started immediately after APS addition. Emission intensities were normalized to the first time point.

### Fluorescence intensity pre vs. post re-embedding

In addition to ensemble measurements, Airyscan images of alpha-tubulin in COS-7 cells were used to analyze the fluorescence intensity of the fluorophore AF647. Using the *d*TREx protocol, images of the same cell before and after re-embedding in the neutral gel with 0.025% APS and 0.025% TEMED were taken. ROIs of identical tubulin strands were selected, and an auto threshold (“Otsu”) was applied. After converting the image to a mask, the outline of the mask (“Create Selection”) was saved in the ROI manager. This ROI was then applied to the original image, and the mean fluorescence intensity in this region was measured.

### Evaluation of timepoint for adding secondary antibodies

The potential different effect of adding secondary antibodies (sAbs) before (pre) or after (post) re-embedding in the neutral gel was analyzed. Therefore, *d*TREx gels labeled for α-tubulin were labeled with identical secondary antibody concentrations and incubation times either pre or post re-embedding into the neutral gel. In gels with sAbs added post re-embedding, washing with PBST was conducted more thoroughly (5 × 1 h) with an additional overnight washing step with gentle agitation. In contrast, in gels with sAbs added before re-embedding, washing with PBST was done as in the standard protocol (3 × 15 min). Airyscan images were recorded with the same settings for both conditions, and the fluorescence intensity of microtubule strands was evaluated. The microtubule structure in overview images was defined in the same way as described in the previous section (“Fluorescence intensity pre vs. post re-embedding”). Then the fluorescence intensity in the applied ROIs to the original images was measured. Further, three ROIs in each overview image were drawn to measure the background fluorescence intensity. The intensity was then normalized to the ROI area for comparison of the two different conditions.

### Expansion factor determination

The expansion factor was calculated by comparing Airyscan images of identical areas in the samples before and after expansion. On the one hand, landmarks (e.g., the distance between the same two clathrin particles) were measured (as shown in Ext. Data Fig. 1e). In addition, the expansion factor was determined using a custom-written Python script as described before^69^. Initially, images of the identical structure in the unexpanded (imaged with 63× oil objective) and expanded state (imaged with 10× or 20× objective) were registered via a rigid similarity transformation, providing the structural expansion factor. Optionally, a Gaussian blur was applied to expanded images to better match the resolution of the unexpanded image and facilitate the registration. Further, a non-rigid affine transformation was implemented, which is used to calculate the difference to the similarity transformation, representing nonlinear distortions depicted in a distortion vector map. The same script was used to determine the loss of expansion during the re-embedding process for *d*STORM.

### CCP quantification

To evaluate the expansion efficiency of different protocols, the diameter of CCPs was measured using 3D Airyscan images and Fiji. The center of each individual CCP was selected by going through the z-stack. A line profile was drawn over the CCP, and an intensity profile was generated. The diameter was determined by measuring the peak-to-peak distance in the resulting plot. If only one peak was observable, the FWHM was measured.

### Cluster quantification of AP2 and clathrin

In 2-color Ex-*d*STORM images of AP2 and clathrin, clusters were classified and counted using DBSCAN and a custom-written Python script using LOCAN^57^. DBSCAN parameters were epsilon = 20, meaning all points that lie in maximum distance of 20 nm to the next point are grouped into a cluster. All points that are reachable from at least 3 points (minPts = 3) belong to the cluster core. If a point is only reachable by one other point, it is still included in the cluster as a border point. Thresholds of 80 photons for the AF647 channel and 400 photons for the CF568 channel were set. Radial distribution functions for Ex-*d*STORM localizations were carried out with Python scripts based on the LOCAN package. The procedure for radial distribution analysis of Ex-*d*STORM localizations and modeled antibody epitopes from pdb-structure 1XI4 is visually explained in Supplementary Figs. 13–15.

### LineProfiler measurements

For comparing the expansion efficiency of filamentous microtubule structures after undergoing four different expansion protocols, LineProfiler was used to determine the average diameter of microtubules^70^. Airyscan images of microtubules were recorded using the same settings for all conditions. During data processing, the pixel size was set to 65 nm. To compensate for noise or incomplete data, the following parameters were applied: intensity threshold of 2, spline interpolation parameter of 3, and a blur radius of 20. These configurations affect only the positioning and orientation of the line profiles and do not alter the underlying image data. For each condition, a total of 270–550 line profiles were fitted with a Gaussian function, and the full width at half maximum (FWHM) of the fits was used to determine the overall diameter of the microtubules. For graphical illustration, the line profiles were normalized to [0, 1], centered and overlayed.

### NPC quantification

Sizes of NPCs immunostained for NUP96 in *d*TREx gels were evaluated by manually measuring their maximum diameter using a line profile and its peak-to-peak distance in Ex-*d*STORM images of 9 different nuclei. Furthermore, in these images, potential NUP96-dimers were selected manually based on their appearance and their distance was measured with a line profile and its peak-to-peak distance.

### Statistical analysis

All Ex-*d*STORM experiments were repeated at least three times (3 independent experiments), except for AP2/Clathrin two-color measurements (2 independent experiments) and NPC measurements (one experiment), with comparable results. Data was analyzed using the software OriginPro (Version 2021b, OriginLab Corporation). Scatter dot plots, as well as their descriptions in the text, show the mean value ± standard deviation (s.d.). The number of objects analyzed are represented as single data points in the graphs. For testing normality, the Kolmogorov–Smirnov test was conducted. Significant differences between the mean values of two groups were determined with a two-sample t-test. For two connected samples, a paired sample t-test was conducted. Significant differences between the mean values of more than two groups were tested using a one-way ANOVA with a subsequent Tukey test. *P*-values illustrated as * ≙ *p* < 0.05, ** ≙ *p* < 0.01, *** ≙ *p* < 0.001, **** ≙ *p* < 0.0001 and ns ≙ non-significant.

### Analysis of microtubule distances

We manually selected straight microtubule segments between microtubule intersections, automatically determined their 3D centerline based on cluster coordinates, and analyzed the axial distances of localizations in angular segments.

To determine the central axis of MTs, we first detected clusters of localizations using *hdbscan* and then calculated the average position of the 10 nearest neighbors of each cluster. These average positions lie near the central axis of the MT and were thus used as initial center points. For each cluster, we calculated the distance to the nearest center point, and all clusters further away than a distance of 250 nm from the nearest center point were discarded. The procedure was then repeated with the remaining clusters. The final axial centerline was determined by fitting a bicubic spline (*k* = 3) through the detected centerline points.

### Axial coordinate system

The centerline was used to define a microtubule-local coordinate system for all localizations and cluster centers. Each localization/cluster is defined by its axial position along the MT, its angle measured counter-clockwise relative to 12:00, and its radial distance from the MT axis.

### Distance analysis

Due to the angular offset between the protofilaments constituting the microtubule, no periodicity along the axis can be detected when averaging over all angular positions, since all axial distances would be equally probable. However, when moving along the MT axis at a given angular position, tubulin dimers are spaced 8 nm apart, and thus a periodicity of multiples of 8 nm (and multiples of 4 nm around the seam) should be detectable. We therefore calculated the axial autocorrelation function of a Gaussian-smoothed histogram (*σ* = 2.5 nm) of all cluster positions along the MT axis separately for 16 angle bins to detect the periodicity of the microtubule lattice, followed by averaging over all angle bins.

### Simulation

To calibrate the analysis and to compare the results to theoretical expectations, we simulated MTs with α-β tubulin heterodimers stacked axially in 13 parallel protofilaments with a helical offset of 12 nm. A given fraction of α-tubulin monomers was randomly selected as labeled with a primary antibody. An additional offset of 37.5 nm (3 × 12.5 nm) in a random direction due to the linkage error after the second expansion and a localization uncertainty of 2 nm (xy) and 5 nm (z) (unexpanded) was used to define the final positions of the detected cluster centers. The labeling efficiency was estimated by comparing the number of detected cluster centers to the number of α-tubulin monomers per MT length. We then performed the same distance analysis as described above using the simulated emitter positions as cluster centers.

### Ex-*d*STORM data analysis for two-color experiments in neurons

Ex-*d*STORM data from Munc13-1/RIM two color measurements resulted from three independent experiments for each condition, respectively. Using the localizations determined with rapidSTORM 3.3 (as described above), we calculated and displayed Ripley’s H-function, a normalized Ripley’s K-function. Computation was carried out for each ROI without edge correction. The averaged H-function and a 5–95% confidence interval was computed from 100 simulated data sets with localizations distributed on identical ROIs with identical number of localizations in each ROI according to complete spatial randomness or a Neyman–Scott process. The Neyman–Scott clustering process resembles typical *d*STORM data. It has homogeneously distributed parent events with each parent having n offspring events, where n is Poisson distributed with mean 10, and with the offspring positions having a Gaussian offset with a standard deviation of 22 nm. The maximum of the H-function indicates a distance that is between cluster radius and diameter and thus provides an estimate for the average cluster size. Localization clusters that contain larger ring-like localization clusters were determined by an arbitrary but reproducible algorithm to compare cluster features between the various treatment conditions. Clusters were determined by combining all Munc13-1 and RIM localizations, running a DBSCAN algorithm with epsilon = 100 nm and minPoints = 20, and selecting those clusters with a convex hull area between 100,000 and 500,000 nm^2^, a isoperimetric quotient larger than 0.85, and a radial distance between 150 and 200 nm. All analysis procedures were carried out with Python scripts based on the LOCAN package.

### Estimation of Munc13-1 and RIM diameters in active zones

The sizes of RIM and Munc13-1 substructures in reconstructed Ex-*d*STORM images were evaluated in Fiji. The polygon tool was used to outline the outer border of the assembly of ring-like structures (Supplementary Fig. 21). Subsequently, Feret’s diameter was determined to obtain the maximum diameter of the structure. The average diameter of ring-like structures in expanded active zones of hippocampal neurons was determined to a mean of 403 nm (Munc13-1) and 437 nm (RIM), independent of the experimental conditions (Fig. 4d, e). Since proteins are immunolabeled with a primary antibody after the first expansion step of the *d*TREx protocol, we added 12.5 nm as an average size value for a primary IgG antibody. The primary antibody is digested, and ~3-fold expanded in the second expansion step, resulting in a displacement of ~37.5 nm. The secondary IgG antibody was neglected as it could potentially bind in any direction and will not be expanded itself. Hence, we subtracted a value of ~75 nm (37.5 nm × 2 for both sides of the ring-like arrangement) from the measured diameters. This results in corrected average diameters of state IV of 328 nm (Munc13-1) and 362 nm (RIM). Divided by the overall expansion factor of 7.5×, we obtain average ring diameters of ~44 nm for Munc13-1 and ~48 nm for RIM rings in state IV, independent of the experimental conditions.

### Reporting summary

Further information on research design is available in the Nature Portfolio Reporting Summary linked to this article.

## Supplementary information

Supplementary Information

Reporting Summary

Transparent Peer Review File

## Source data

Source Data

## Data Availability

Source data are provided with this paper. The raw data and source code generated in this study have been deposited in the Zenodo database under 10.5281/zenodo.21290042. Source data are provided with this paper.

## Code availability

The raw data and source code generated in this study have been deposited in the Zenodo database under 10.5281/zenodo.21290042.
