# Supplementary material for: Resolving endogenous protein organization in cells with nanometer resolution
Source: Nat Commun. 2026 Jul 29;17:7583. doi: 10.1038/s41467-026-76146-7 (PMC13421506; doi:10.1038/s41467-026-76146-7)
Supplement: Supplementary file 1 — Supplementary Information [file 41467_2026_76146_MOESM1_ESM.pdf]

Supplementary Information

Resolving endogenous protein organization in cells with nanometer resolution

Janna Eilts[1], Marvin Jungblut[2], Dominic A. Helmerich[1], Stefan Sachs[1], Christian Werner[1], Cristian-Alexandru Bogaciu[3], Ali H. Shaib[3], Silvio O. Rizzoli[3], Philip Kollmannsberger[1], Sören Doose[1] & Markus Sauer[1,2]

[1]Department of Biotechnology and Biophysics, Biocenter, University of Würzburg, Am Hubland, 97074 Würzburg, Germany

[2]Rudolf Virchow Center, Research Center for Integrative and Translational Bioimaging, University of Würzburg, Josef-Schneider-Str. 2, 97080 Würzburg, Germany

[3]Department of Neuro- and Sensory Physiology, University Medical Center Göttingen, Göttingen, Germany

*Corresponding author: m.sauer@uni-wuerzburg.de

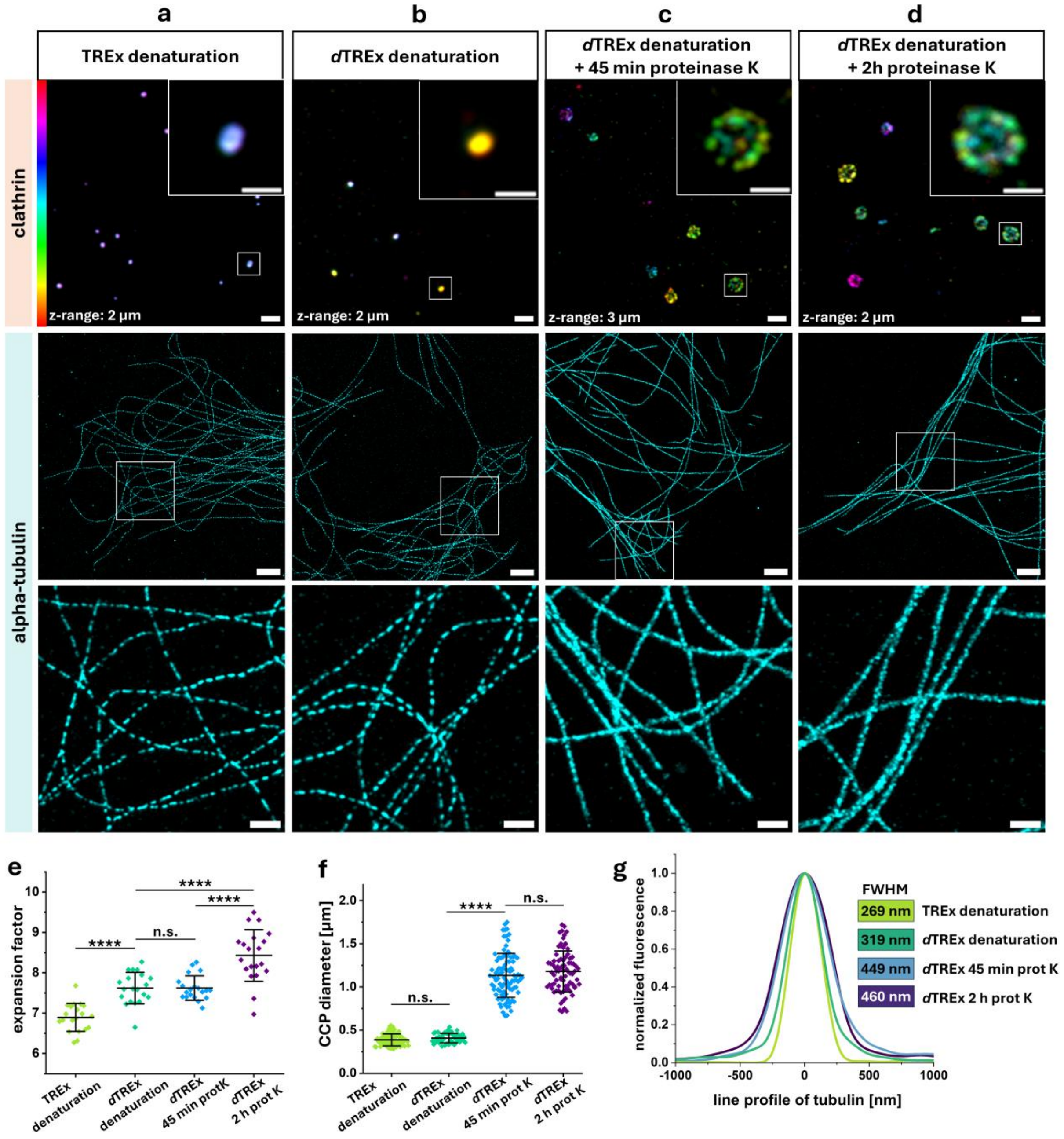

Supplementary Fig. 1. Comparison of different post-immunolabeling approaches. **a-d**, Airyscan fluorescence images of GA fixed and anchored COS-7 cells post-labeled after denaturation for clathrin heavy chain (upper row, scale bars 2 µm; magnified regions 1 µm) or alpha-tubulin (middle row, scale bars 10 µm and lower row for magnified regions, scale bars 3 µm) and processed according to the different protocols. Color code for clathrin images shows the z-range. **a**, TREx with denaturation; **b**, *d*TREx with denaturation; **c**, *d*TREx with denaturation plus 45 min proteinase K digestion at 37°C and **d**, *d*TREx with denaturation plus 2 h proteinase K digestion at 37°C. **e**, Expansion factors of different protocols determined by measuring the distances between landmarks, i.e., the same two CCPs before and after expansion. Data from one experiment using the same monomer solution for all conditions. **f,** CCP diameters using the different protocols. Data from three (TREx denaturation, n = 75; *d*TREx 45 min prot K, n = 94), two (*d*TREx 2 h proteinase K, n = 81) independent experiments and one experiment (*d*TREx denaturation, n = 50). **g**, Line profiles of single microtubule strands using different protocols measured by LineProfiler[68]. The full width at half maximum (FWHM) of Gaussian fits was determined for each curve. Scatter dot plots show mean (line) ± s.d. (whiskers) and single data points (dots). P-values of one-way ANOVA with post-hoc Tukey-test are illustrated as **** ≙ $p < 0.0001$ and ns ≙ $p > 0.05$ (non-significant).

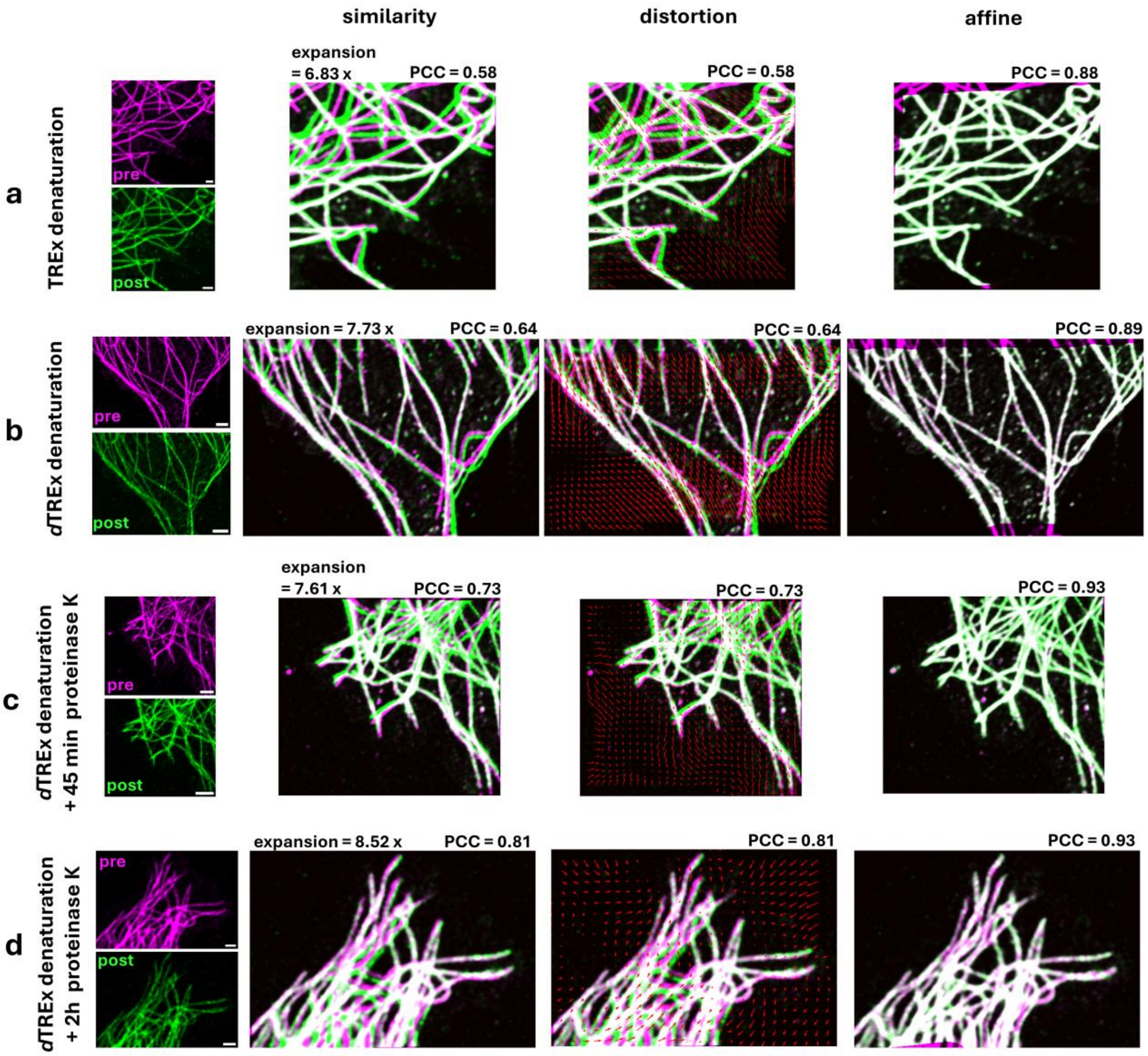

Supplementary Fig. 2. Expansion factors of different protocols in COS-7 cells stained for alpha-tubulin. Cells were fixed and anchored with GA. Denaturation was done with SDS and DTT at 98°C. In (c) and (d) proteinase K was applied at 37°C. Airyscan fluorescence images of the same area imaged pre (magenta) and post (green) expansion. Similarity transformation aligns pre- and post-expansion images by rotation, scaling and translation in x and y direction, yielding an expansion factor and PCC value. The distortion vector map was generated from the differences between similarity and non-rigid affine transformation, which usually yields a higher PCC. **a**, TREx with denaturation. Scale bars pre: 1 µm, post: 10 µm. **b**, *d*TREx with denaturation. Scale bars pre: 2 µm, post: 20 µm. **c**, *d*TREx with denaturation and 45 min proteinase K. Scale bars pre: 2 µm, post: 20 µm. **d**, *d*TREx with denaturation and 2 h proteinase K. Scale bars, pre: 1 µm, post: 10 µm. Scale bars show expanded dimensions.

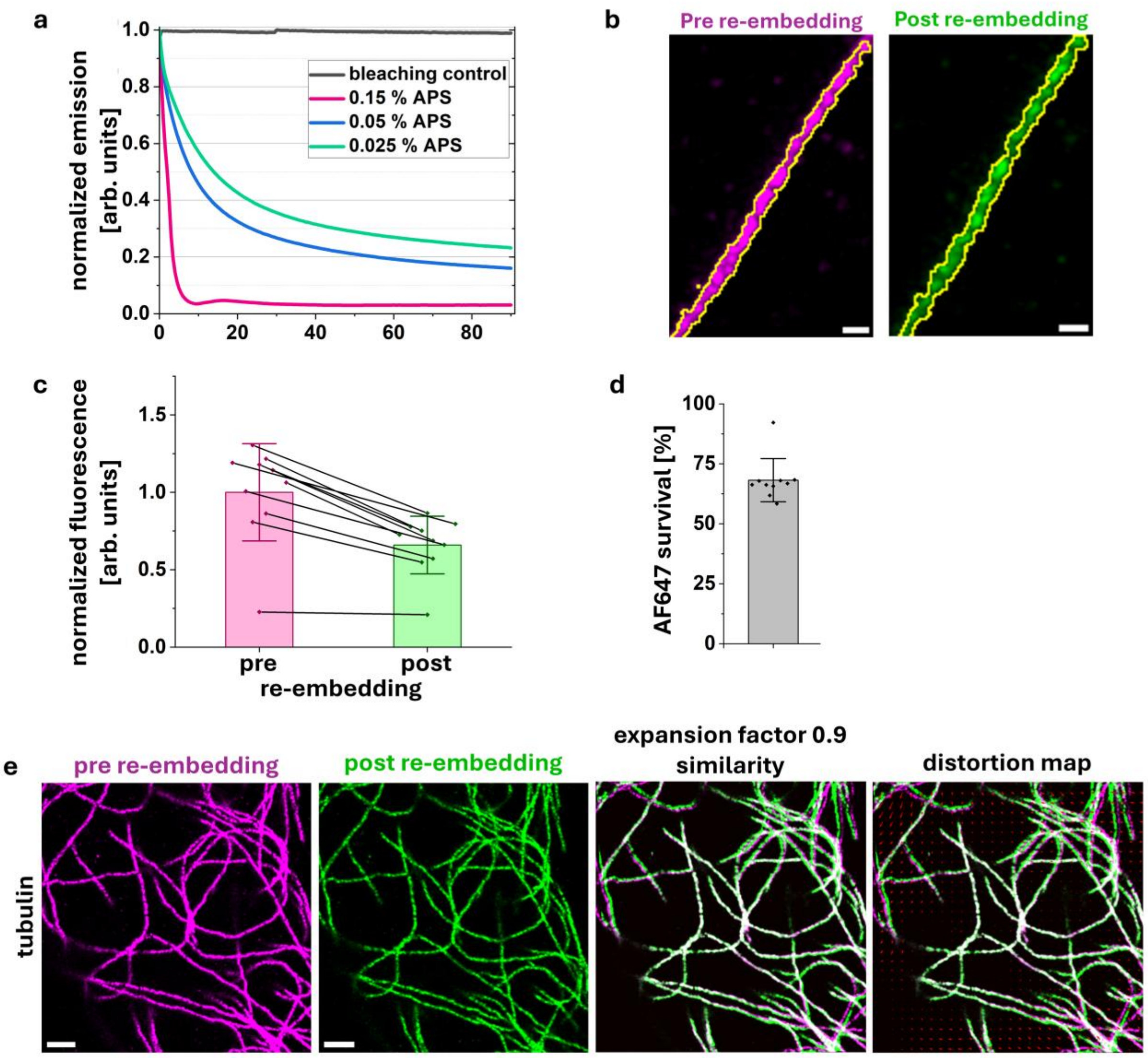

Supplementary Fig. 3. AF647 survival and gel shrinkage during re-embedding in a neutral gel. a, AF647 ensemble emission intensity upon addition of APS and TEMED to an AF647-labeled antibody solution. After 90 min the normalized emission decreased to ~3 %, ~16 % and ~23 % using concentrations of 0.15 %, 0.05 % and 0.025 % APS/TEMED respectively. **b**, Representative Airyscan images of an identical microtubule filament stained with AF647 coupled secondary antibody and imaged before (magenta) and after re-embedding (green). Yellow lines indicate ROI for fluorescence intensity measurement. **c**, AF647 fluorescence intensity of tubulin ROIs (n = 10) normalized to the mean pre-re-embedding intensity. Lines indicate the fluorescence intensity loss for single data points. Data obtained from repeated measurements of one sample. **d**, Mean AF647 fluorescence survival (68 ± 9 %). **e**, Gel shrinkage during re-embedding. Identical areas of alpha-tubulin in COS-7 cells imaged pre (magenta) and post re-embedding (green). Images registered via similarity transformation yield an expansion factor of 0.9. A distortion vector map shows the difference to an affine transformation. Bar graphs show single data points, mean value ± s.d. Scale bars, 1 µm (b), 5 µm (e). Scale bars show expanded dimensions.

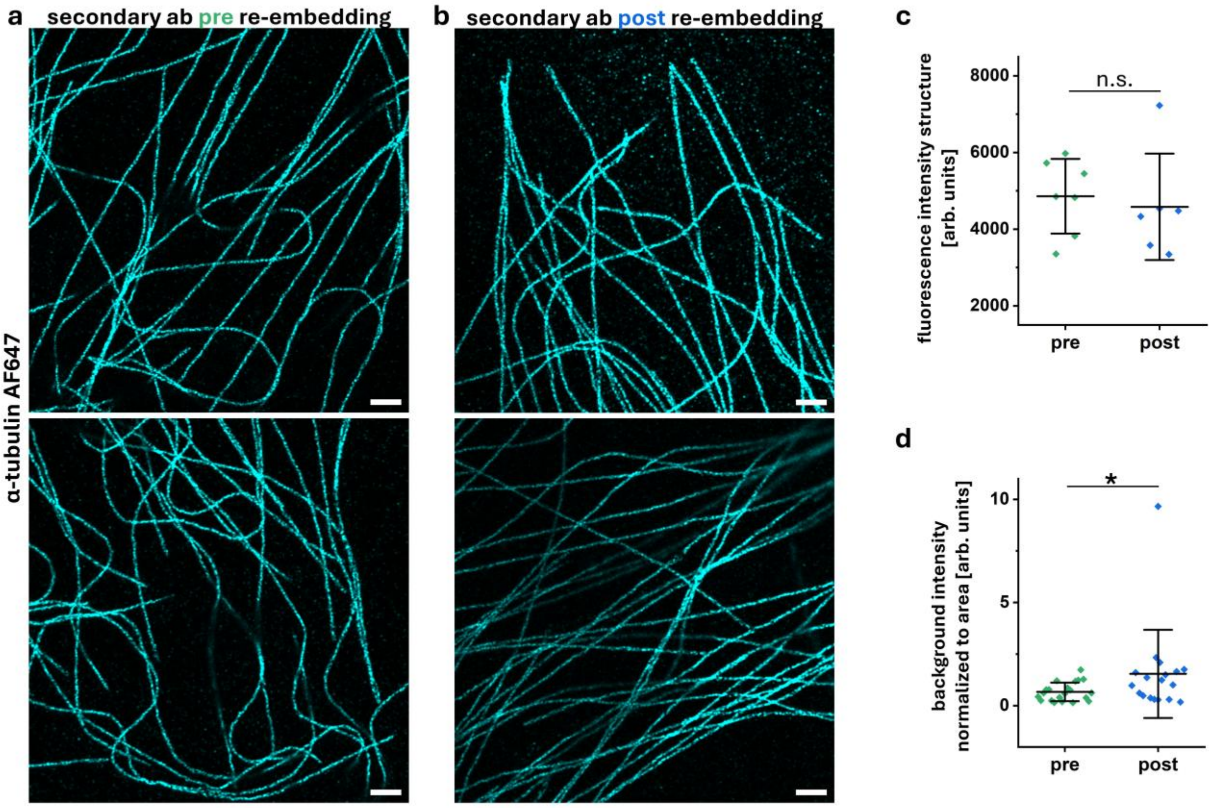

Supplementary Fig. 4. Comparison of pre- and post-re-embedding labeling with secondary antibody. a,b, Representative Airyscan images of *d*TREx gels immunostained for α-tubulin and labeled with AF647-conjugated secondary antibodies either before (**a**) or after (**b**) re-embedding in neutral gel. **c,** Images of microtubules labeled either before or after re-embedding with the secondary antibody show similar fluorescence intensities (p = 0.68 determined by two-sample t-test). Data obtained from n_{pre} = 7 and n_{post} = 6 images from one experiment. **d,** Background signal intensity of microtubule images labeled after re-embedding with the secondary antibody is slightly higher (n_{pre} = 21, n_{post} = 18 ROIs, p = 0.047 determined by Mann-Whitney-U test). Scatter dot graphs show single data points, mean ± s.d. Scale bars, 5 µm. Scale bars show 8.0x expanded dimensions after re-embedding in the neutral hydrogel.

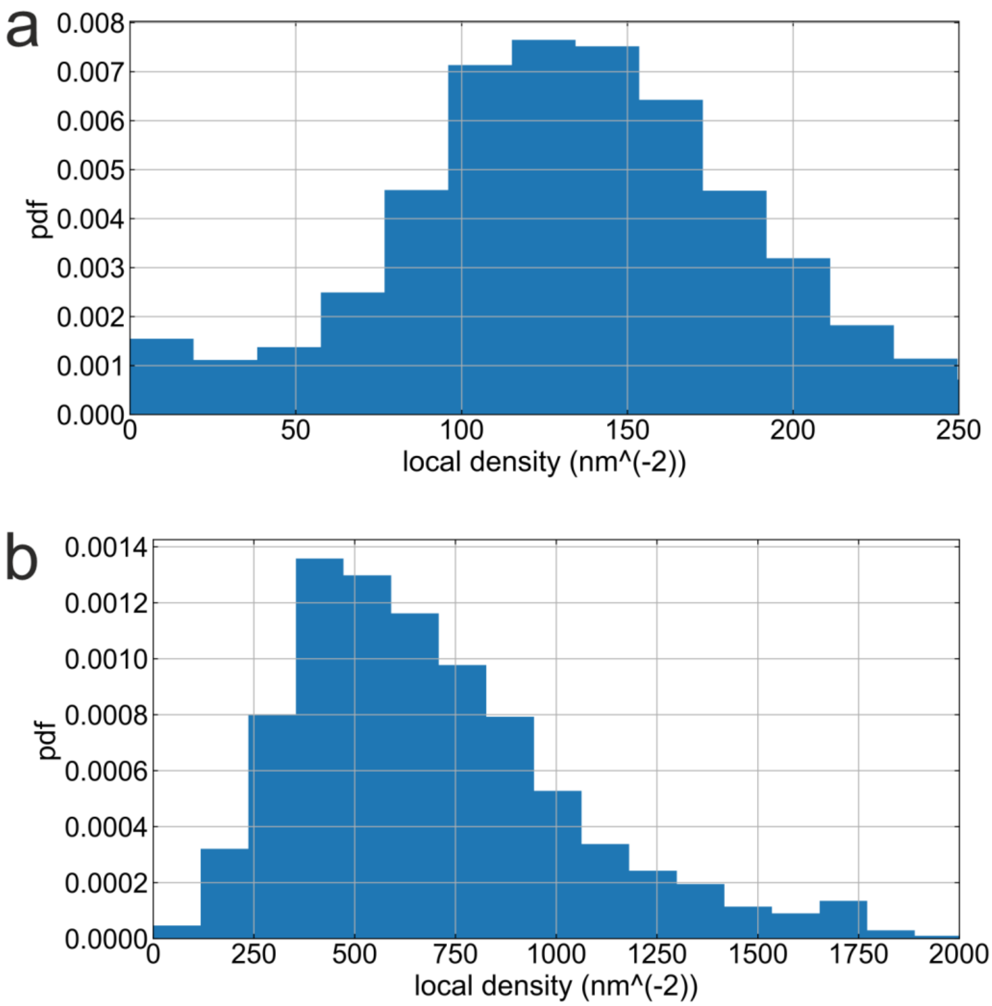

Supplementary Fig. 5. Local densities of *d*STORM localizations for α-tubulin. *d*STORM localizations in successive frames were linked when closer than 100 nm and the local density was computed for each linked localization with the given radius. **a,** Histogram of localization densities (probability density function, pdf) detected for unexpanded microtubules using a 50 nm radius (n=23 ROIs). **b**, Histogram of localization densities detected for ~8-fold expanded microtubules immunolabeled according to the *d*TREx protocol using a corresponding 400 nm radius (n=65 ROIs).

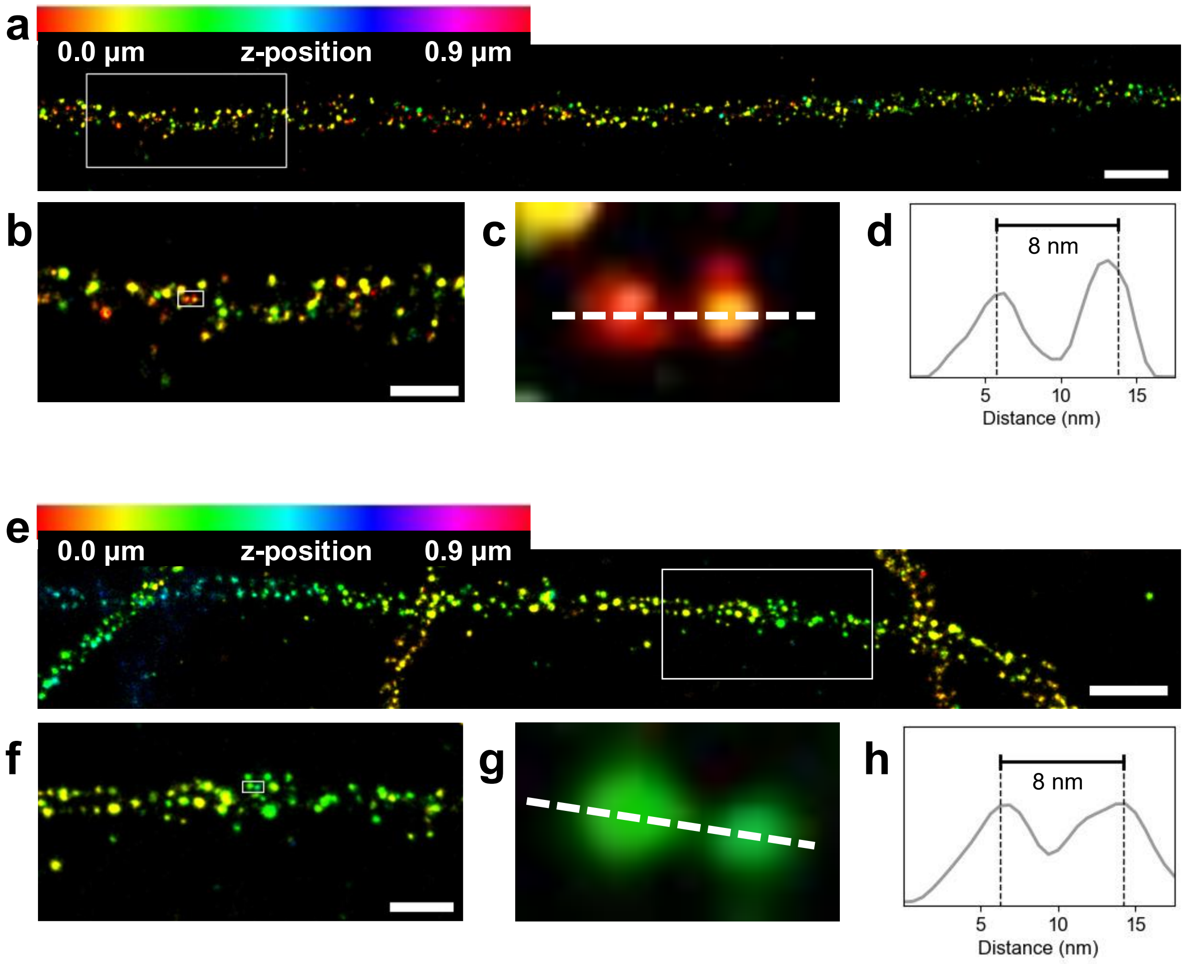

Supplementary Fig. 6. Examples for 8 nm distances between tubulin dimers in cells resolved by Ex-*d*STORM. a,e, Microtubule segments in COS-7 cells imaged using *d*TREx post-immunolabeled for α-tubulin (AF647). **b,f,** Zoomed-in views of regions indicated by the white box in (a) and (e). **c,g,** Zoomed-in view of region indicated by the white box in (b) and (f). **d,h,** Intensity profiles along the dotted lines in (c) and (g), with distances corrected for the final expansion factor of 8.0 after *d*TREx and re-embedding. Scale bars (expanded), a,e, 1 µm; b,f, 300 nm. Pixel size, 5 nm.

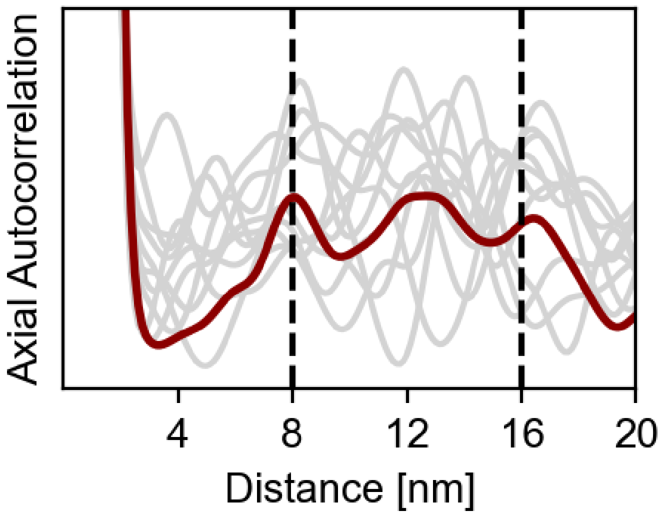

Supplementary Fig. 7. Axial autocorrelation of simulated microtubules using experimentally determined parameters (5% label efficiency, 37.5 nm linkage error). 10 ROIs averaged (dark red) and individual ROIs (light gray).

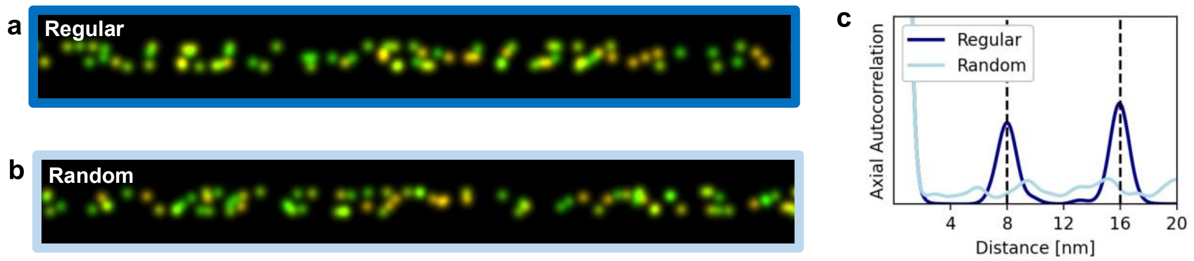

Supplementary Fig. 8. Axial autocorrelation reveals the periodicity of the tubulin lattice. **a,** Simulation of a 8x expanded microtubule segment shown as color-coded z projection, with 5% of α-tubulin labelled, using the parameters extracted from experimental data. **b,** Same data as in (a) with same number of fluorophores, but randomized positions and no underlying lattice periodicity. **c,** Axial autocorrelation of regular (dark blue) and randomized (light blue) tubulin lattice in (a) and (b) reveals the underlying periodicity.

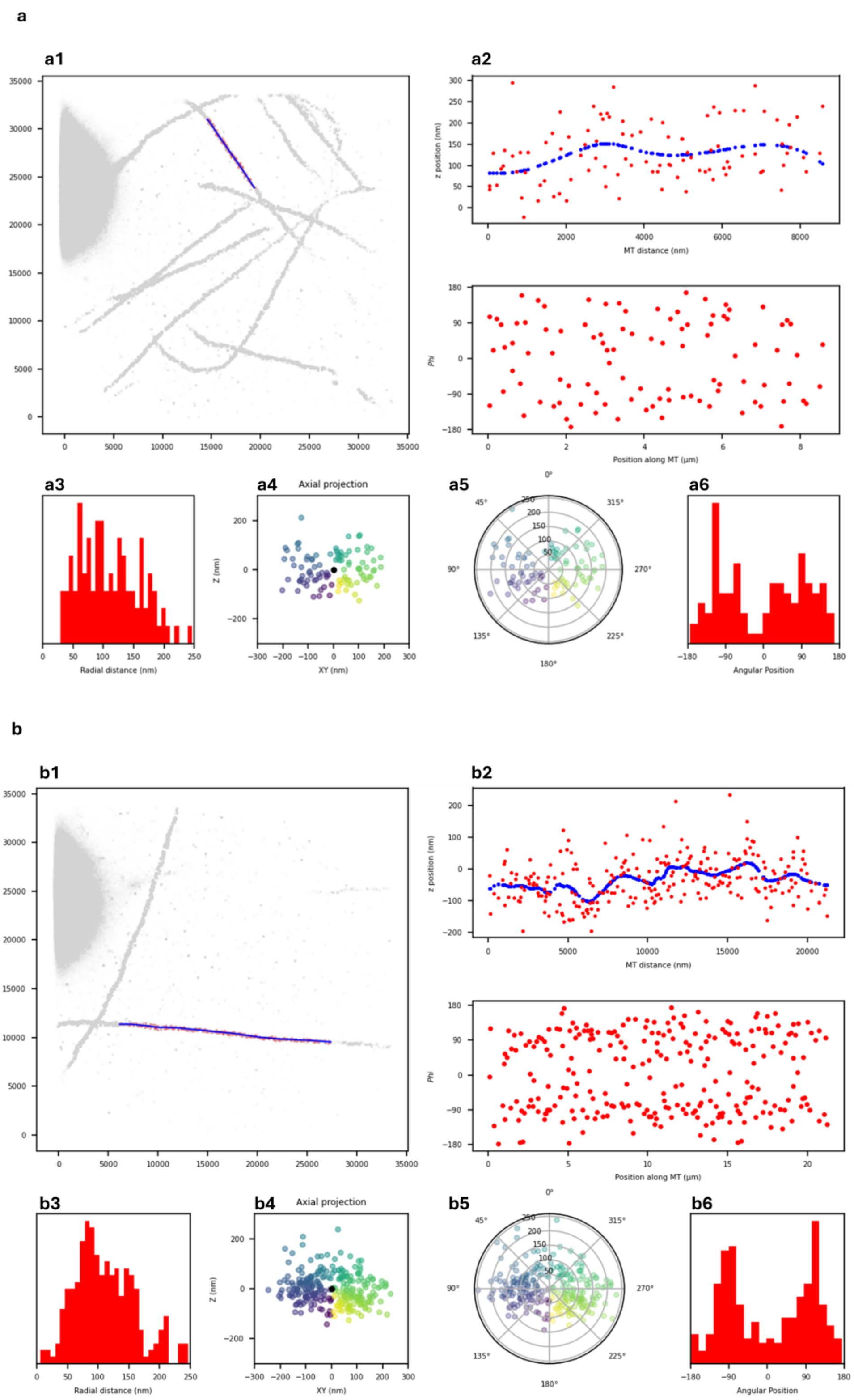

Supplementary Fig. 9. Examples of analyzed regions of interest from Ex-*d*STORM images of microtubules. a1, b1, The blue line indicates the selected microtubule strand. **a2, b2,** Z-position plotted over microtubule distance. The blue line shows the 3D centerline of cluster coordinates (red dots). **a3, b3,** Angular positions of localization clusters (red dots) over microtubule distance. **a3, b3,** Radial distance distribution of localization clusters. **a4, b4,** Axial projection of localization clusters. **a5, b5,** Angular positions of localization clusters as axial projection. **a6, b6,** Distribution of angular positions.

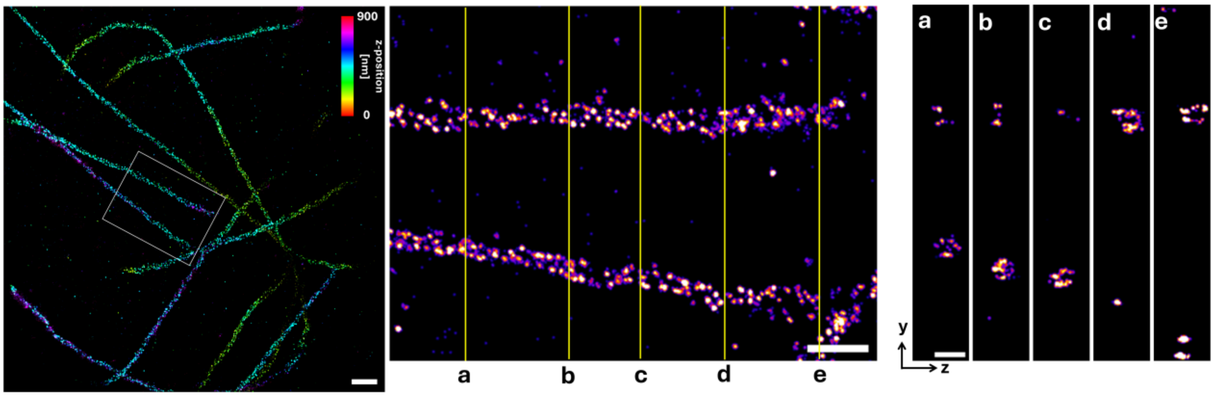

Supplementary Fig. 10. Hollow microtubule cross-sections. Left: Color-coded 3D-Ex-*d*STORM image of microtubules, scale bar 2 µm. White rectangle marks rotated magnified region shown in the middle panel, scale bar 1 µm. Yellow lines in magnified region indicate yz-cross-sections shown in a, b, c and d, scale bar 500 nm.

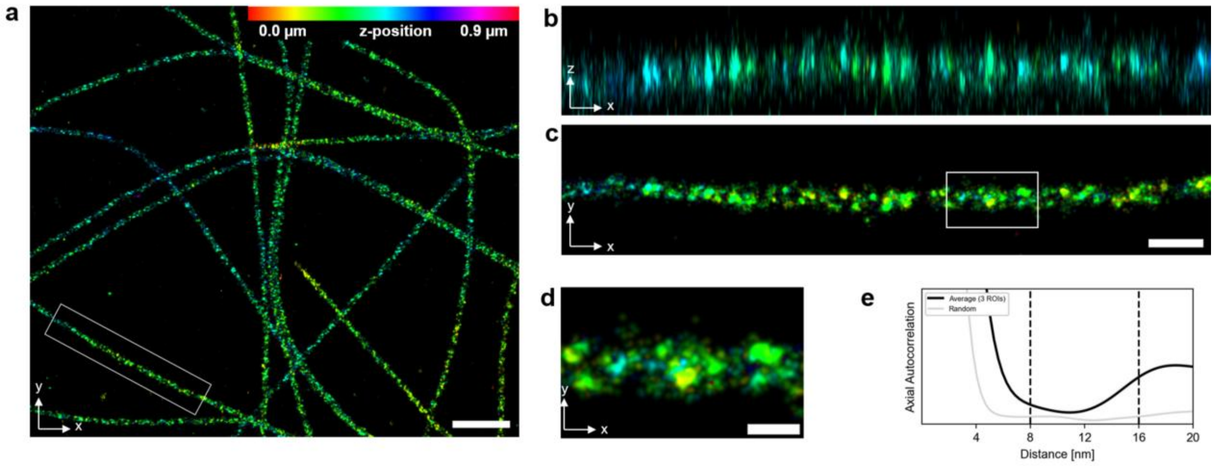

Supplementary Fig. 11. Ex-*d*STORM of 3.2x expanded microtubules does not resolve the 8 nm tubulin lattice. a, Representative 3D Ex-*d*STORM image of re-embedded COS-7 cells expanded ~3.2-fold from Zwettler *et al*. (Ref. 10). **b-c,** Corresponding xz- and xy-views of the region marked in (a). **d,** Zoomed-in view of the region marked in (c). **e,** Autocorrelation function averaged over three microtubule segments (black) and for the same number of clusters randomly placed across the filament (grey). Distance (x-axis) corrected for the expansion factor of 3.2x. Scale bars (expanded), a, 2 µm; b,c, 500 nm; d, 300 nm. Pixel size, 5 nm.

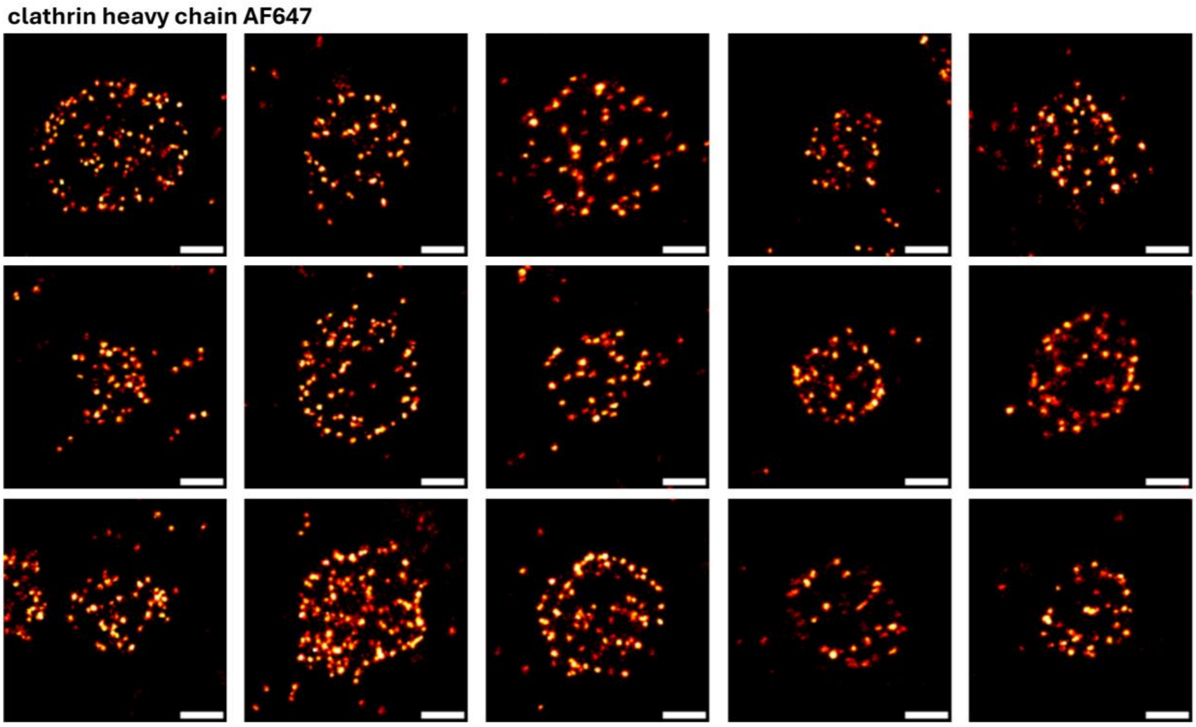

Supplementary Fig. 12. Representative Ex-*d*STORM images of individual CCPs. Gels of GA fixed and anchored COS-7 cells were immunostained for clathrin heavy chain after denaturation with 98°C. Samples were then processed according to *d*TREx including 45 min proteinase K digestion at 37°C. Results from three independent experiments. Scale bars 500 nm (7x expanded dimensions).

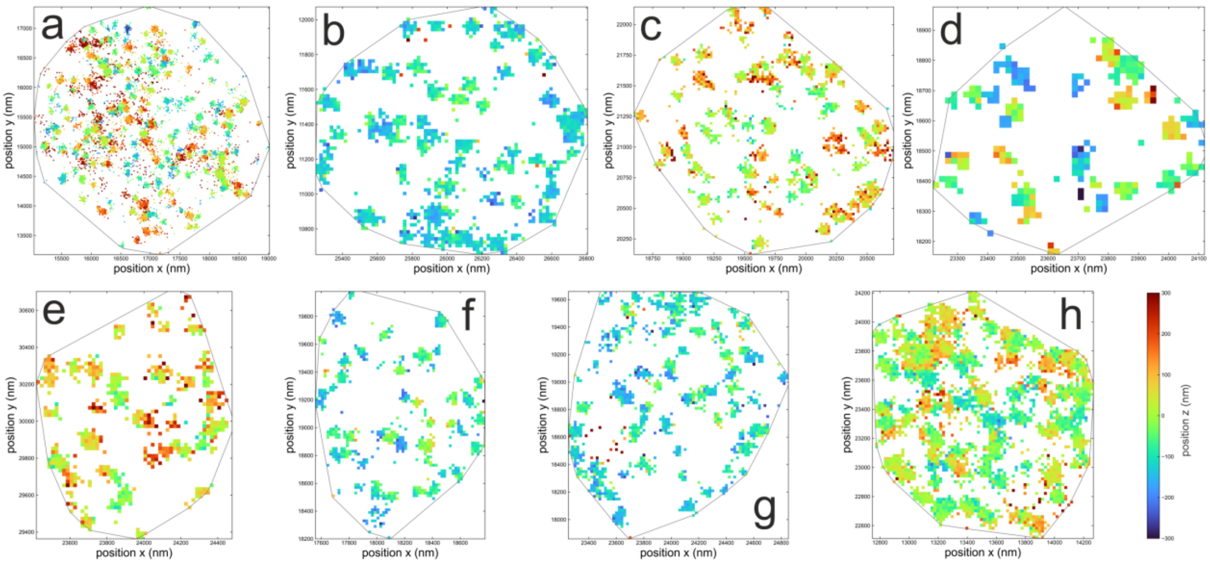

Supplementary Fig. 13. CCPs in 3D-Ex-*d*STORM. Data is shown for the regions of interest that were selected for radial distance distribution analysis in Supplementary Fig. 14. Individual localizations are binned in 20 nm pixels. The turbo color map represents the z-coordinate (average of all localizations per pixel). The gray region represents the 2D convex hull of all localizations projected in the xy-plane.

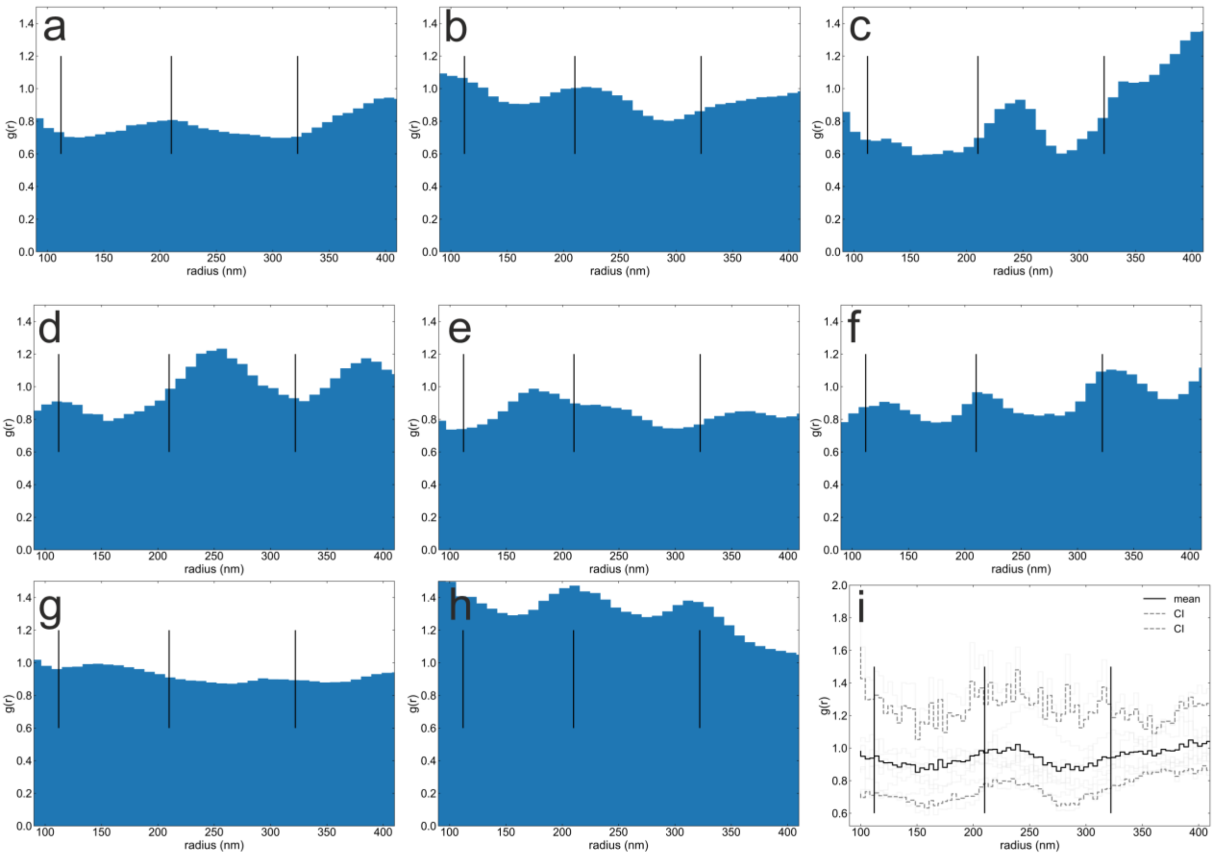

Supplementary Fig. 14. Radial distribution function for individual CCP ROIs. a-h, The radial distribution function is shown for all pairwise localization distances from the recorded *d*STORM localization data shown in Supplementary Fig. 13. **i**, The average radial distribution function with 5/95% confidence intervals for the same rois (same as in Fig. 3g). The vertical lines show expected peak positions. Radial distribution functions are shown relative to those for localizations distributed under complete spatial randomness in identical regions.

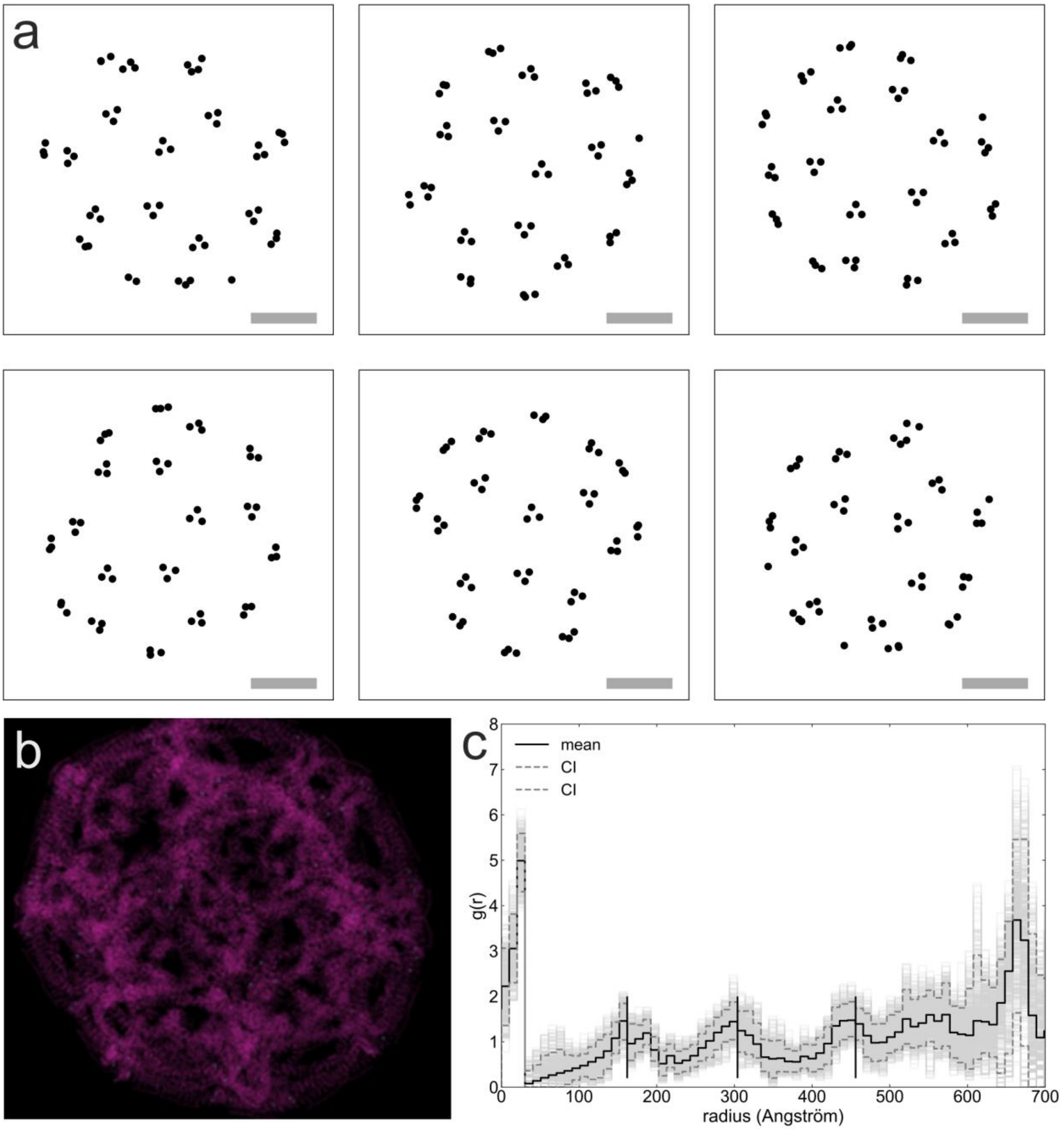

Supplementary Fig. 15. Radial distribution functions for antibody epitope positions in CCP model structure. a,b, Vertices that represent the antibody epitopes on clathrin heavy chain (represented by amino acid 1551) are generated from pdb structure of clathrin D6 coat (pdb: 1XI4). For comparison with experimental data, the vertices on the upper half are selected and randomly rotated in 3D and then projected on the xy-plane. Scale bar: 20 nm. **c,** Radial distribution functions for paired distances are generated and plotted (light gray lines) together with mean (black line) and 5/95 % confidence intervals (dashed gray lines) (n=100). Radial distribution functions are shown relative to a 2D spatial distribution under complete spatial randomness within the convex hull of all points combined.

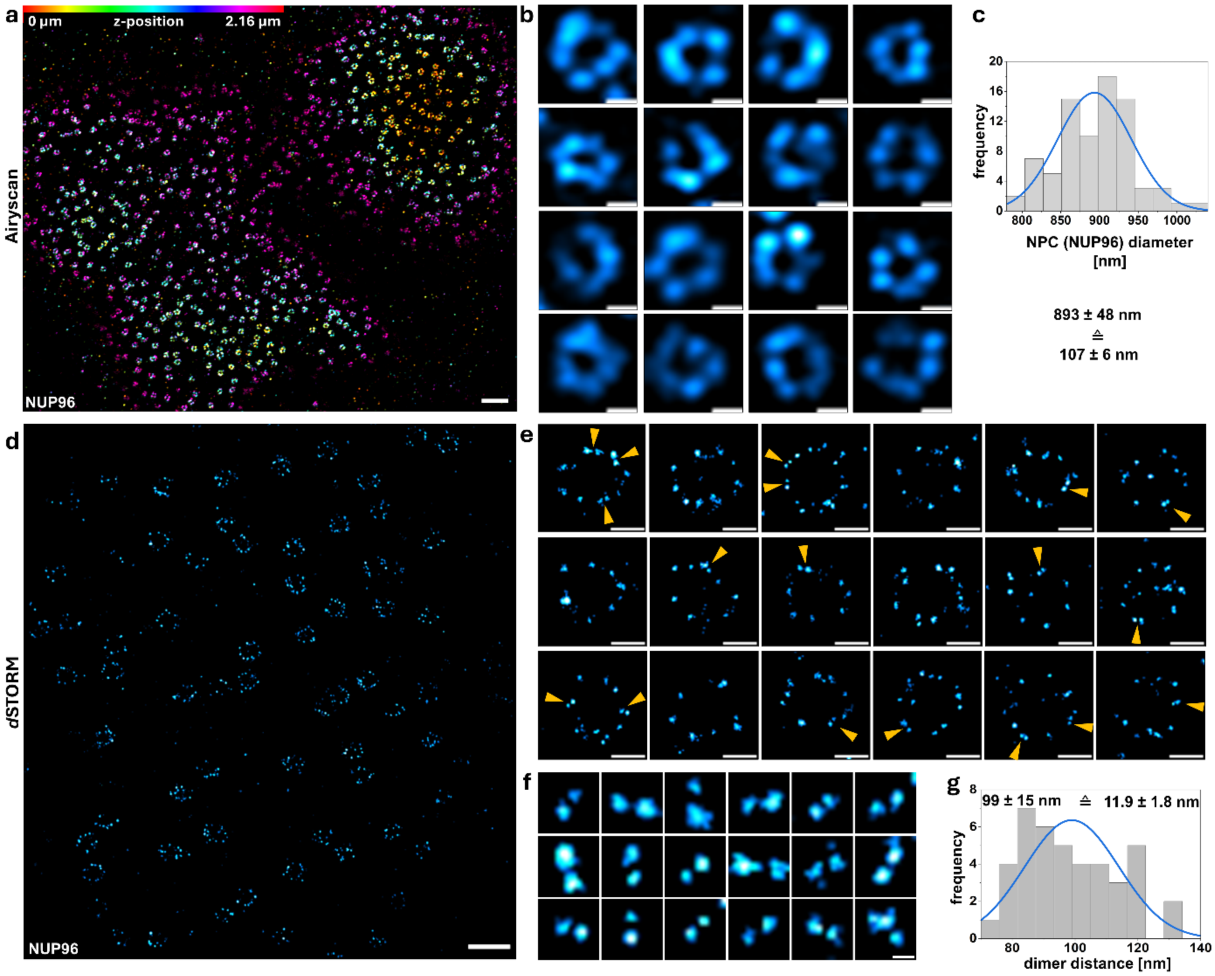

Supplementary Fig. 16. *d*TREx of nuclear pore complexes (NPCs) in COS7 cells. a,b, Airyscan images after re-embedding in the neutral hydrogel. Color-coded z-projection of a nucleus part immunostained for NUP96 (a). Magnified NPCs taken from single slices of Airyscan z-stacks (b). **c,** Histogram of NPC diameters with normal distribution curve (blue) showing an average diameter of 893 ± 48 nm (mean ± s.d., n = 80 from 9 cells from one experiment) in Ex-*d*STORM images. This corresponds to ~8.3-fold expansion after re-embedding considering the known mean diameter of 107 nm for NPCs marked by NUP96[48]. **d-f,** *d*STORM of re-embedded *d*TREx gels showing an overview (d) and magnified NPCs (e) immunostained for NUP96. Yellow arrows indicate NUP96 dimers. **f,** Representative dimer signals used for distance measurements in (g). **g,** Histogram of peak-to-peak distances of manually selected dimers with normal distribution curve (blue) showing an average distance of 99 ± 15 nm (mean ± s.d., n = 41) corresponding to 11.9 ± 1.8 nm considering the previously in (c) determined expansion factor of 8.3. Data from one experiment. Scale bars, 5 µm (a) 0.5 µm (b,e), 2 µm (d) 100 nm (f). Scale bars show 8.3x expanded dimensions after re-embedding

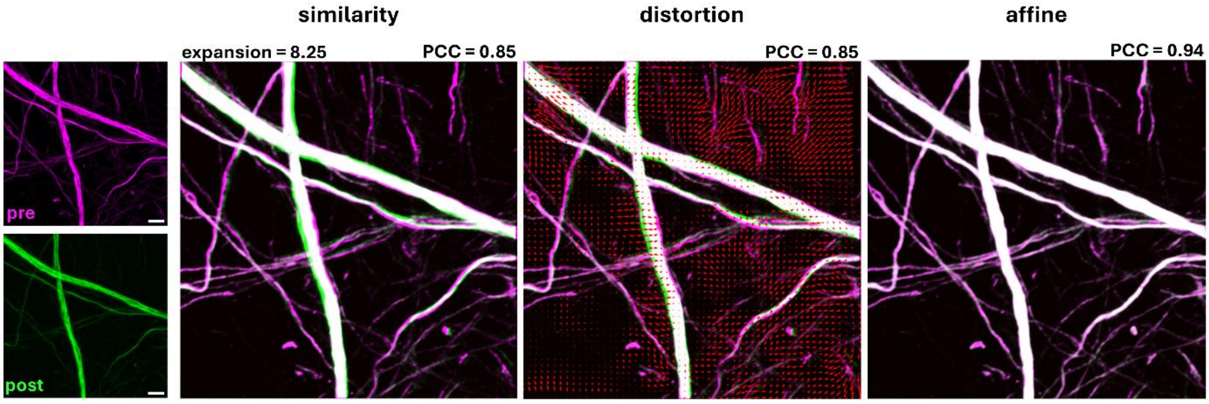

Supplementary Fig. 17. Expansion factor of *d*TREx in neurons. Primary hippocampal mouse neurons were fixed with FA and anchored with FA+AA. Neurofilament-H immunostaining was used to determine the expansion factor of *d*TREx using denaturation at 98°C and 45 min proteinase K digestion at 37°C. Airyscan images of the same area pre-expansion (magenta) and post-expansion (green) were registered by similarity transformation yielding an expansion factor of 8.25x, a PCC value and an affine transformation. The distortion vector map illustrates the differences between similarity and affine transformation. Considering ~10 % shrinking during re-embedding into the neutral gel, the expansion factor was estimated to ~7.5x in Ex-*d*STORM images. Scale bars, 3 µm (pre expansion), 25 µm (post expansion).

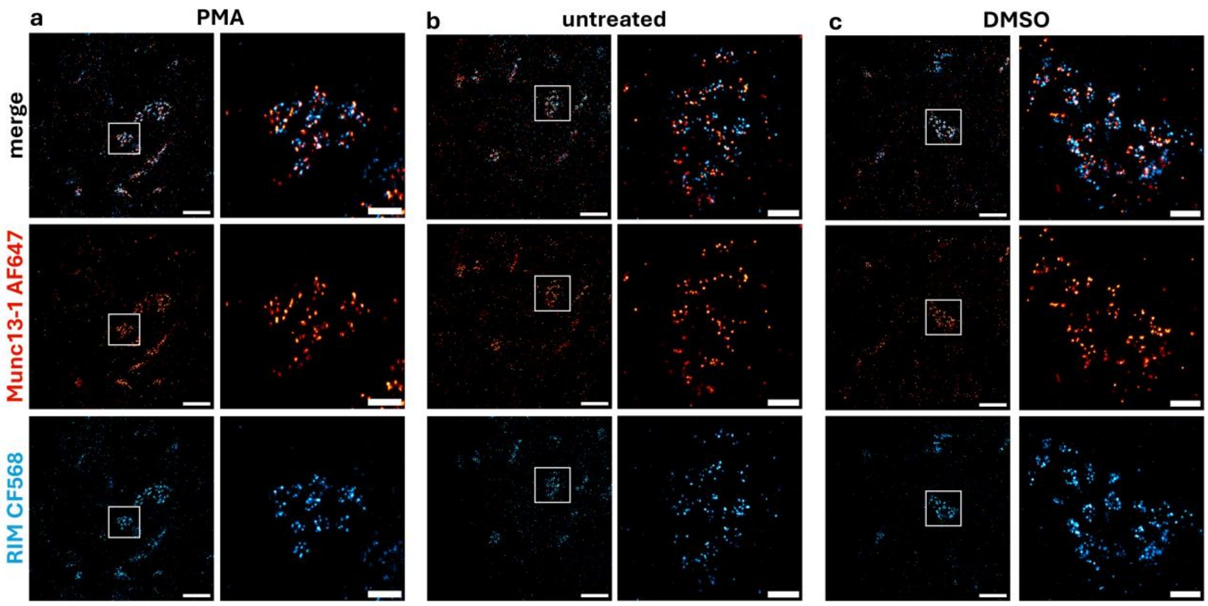

Supplementary Fig. 18. Two-color Ex-*d*STORM images of Munc13-1 and RIM show frontal views of active zones of presynapses. Hippocampal mouse neurons with different treatments before fixation with FA and anchoring with FA/AA, processed by *d*TREx using denaturation with SDS and DTT at 98°C and 45 min proteinase K at 37°C. From overview images a frontal view synapse was selected (white square) and magnified. **a,** Neurons treated with PMA. **b,** Untreated neurons. **c,** Control with DMSO (solvent used for PMA treatment). Overview images scale bars, 5 µm, pixel size, 60 nm; magnified regions scale bars, 1 µm, pixel size, 20 nm. Scale bars show 7.5x expanded dimensions after re-embedding in the neutral hydrogel.

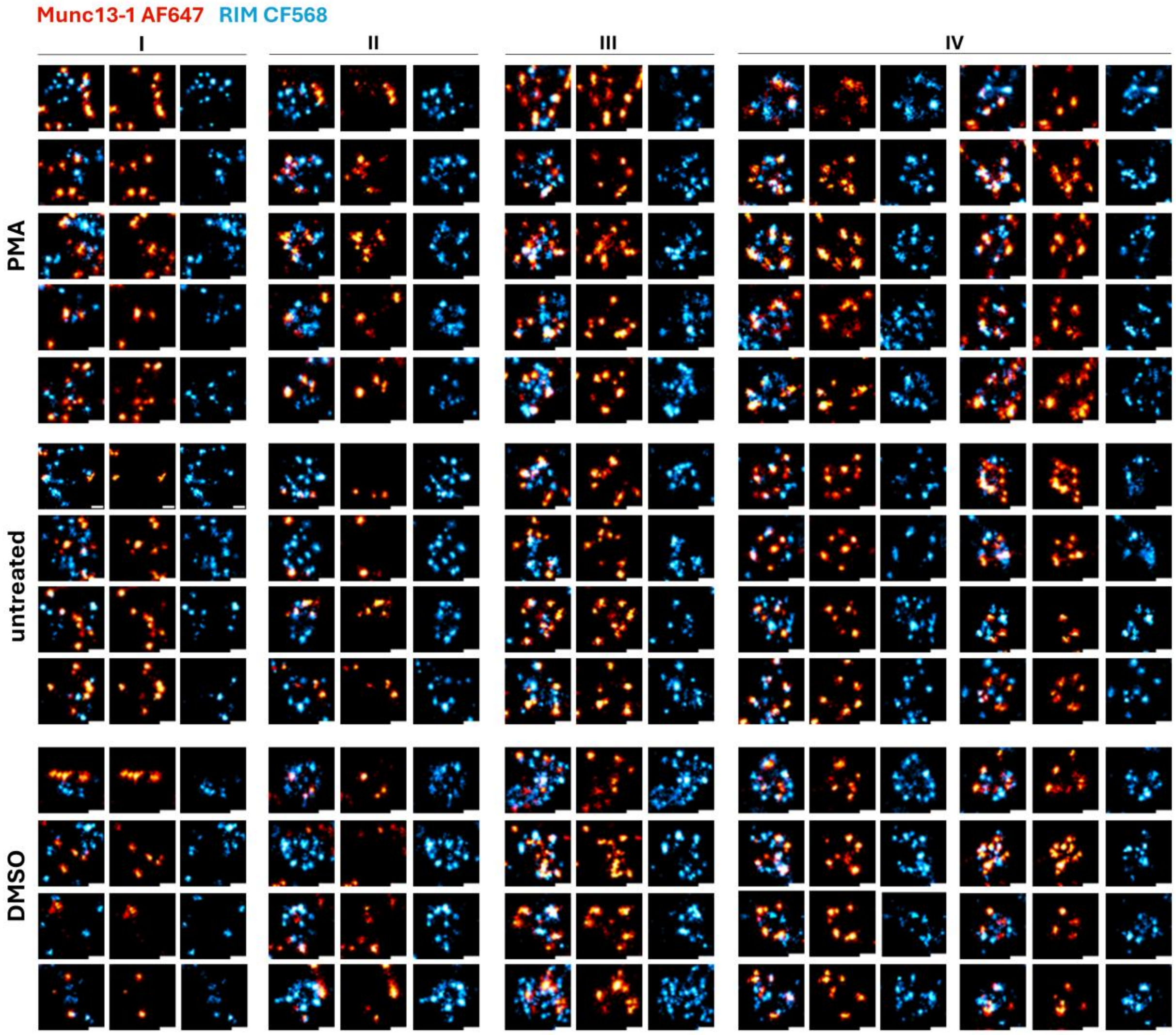

Supplementary Fig. 19. Hand-picked classification of ring-like structures of Munc13-1 and RIM into four classes under different experimental conditions. Magnified Ex-*d*STORM images of individual docking sites show regions of varying sub-structures from different synapses categorized in four different states. I: Munc13-1 and RIM unorganized. II: Only RIM shows ring-like arrangements. III: Munc13-1 and RIM are organized in substructures with a diameter > 500 nm. IV: Munc13-1 and RIM are both organized in ring-like structures with varying diameters. Scale bars, 200nm. Scale bars show 7.5x expanded dimensions after re-embedding in the neutral hydrogel.

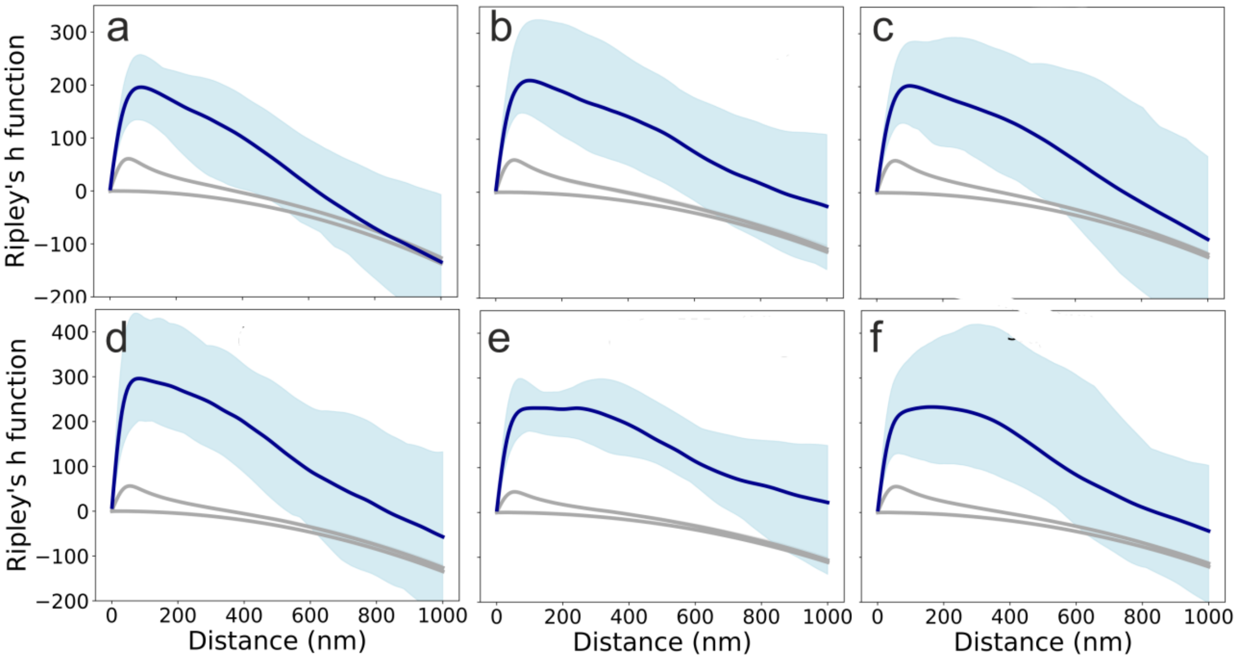

Supplementary Fig. 20. Ripley h-function of *d*STORM localizations recorded for Munc13-1 and RIM immunolabeling. The h-functions for experimental data are shown in dark blue with a 5-95% confidence interval (light blue) from multiple synapses for Munc13-1 (**a-c**) and RIM (**d-e**). The distance refers to expanded samples. Neurons were treated according to the following groups: untreated (**a, d,** n=16), DMSO-control (**b, e,** n=10), PMA treated (**c, f,** n=22).The h-function from simulated data spatially distributed according to complete spatial randomness or according to a clustering process that resembles *d*STORM with homogeneously distributed emitters (grey lines), is outside the experimental confidence interval for length scales up to ~100 nm (~750 nm expanded). This indicates clustering processes distinct from repetitive *d*STORM blinking on different clustering length scales. Scale bars represent ~7.5x expanded dimensions.

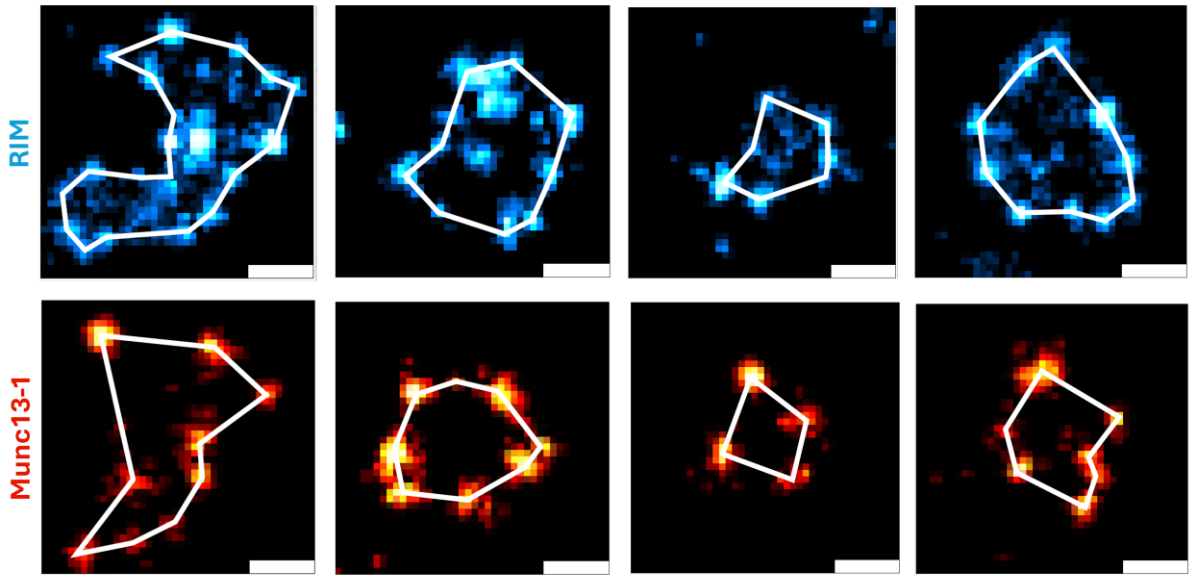

Supplementary Fig. 21. **Size analysis of Munc13-1 and RIM structures in individual synapses.** Synapses and substructures were identified as regions of interest by user selection. Using a polygon tool the outer signals of the respective structure were connected and the Feret's diameter was measured to determine the maximum diameter of the structure. This was done for selected structures of state II (only RIM), state III and state IV shown in Fig. 4b, Supplementary Fig. 19, and additional similar structures. Scale bars, 200 nm (7.5x expanded dimensions).

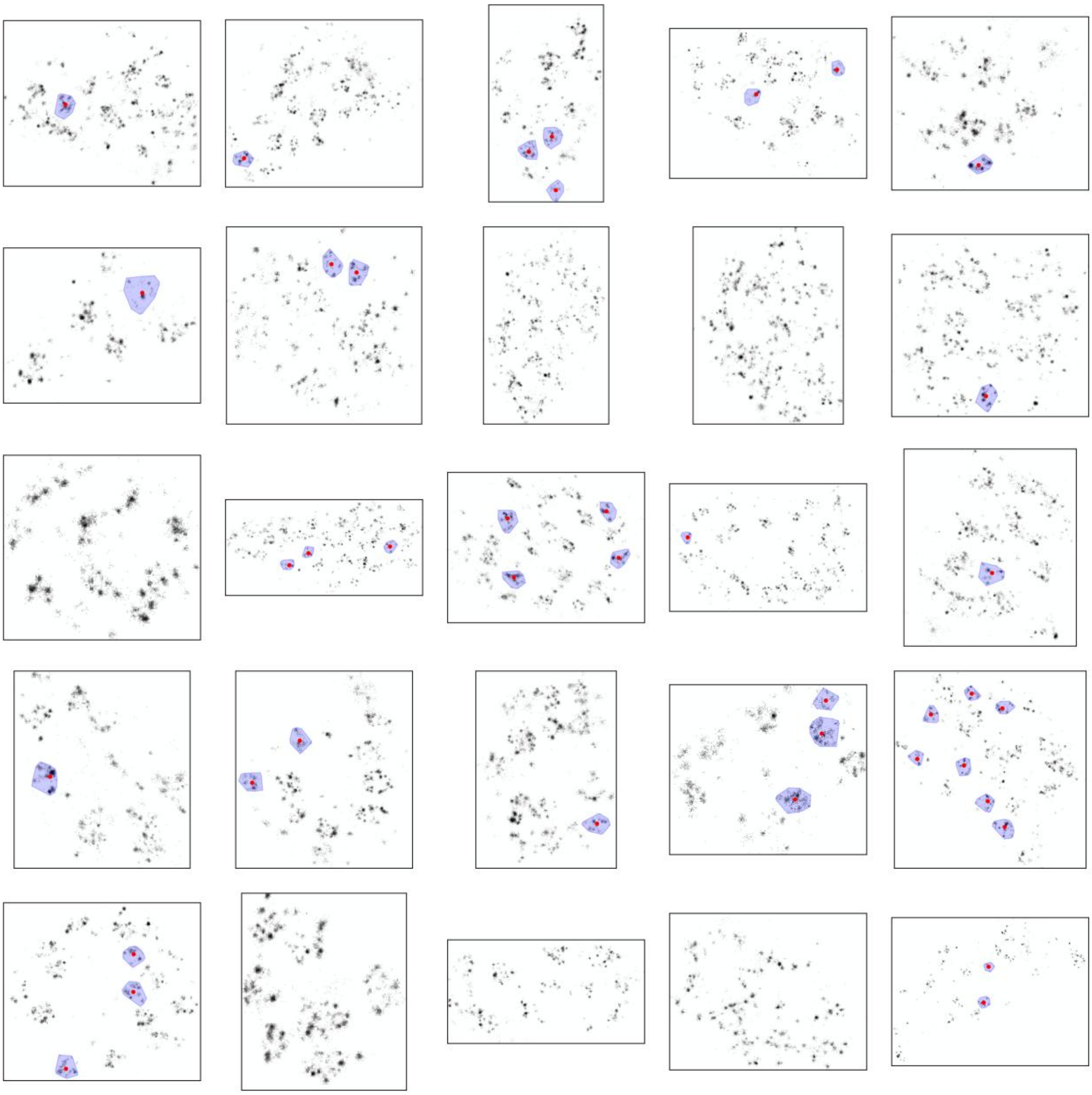

Supplementary Fig. 22. **Cluster identification of Munc13-1 and RIM signals in individual synapses.** Synapses were identified as regions of interest by user selection. DBSCAN was used to identify clusters of the combined set of Munc13-1 and RIM localizations within each synapse. A reproducible set of clusters was selected based on convex hull areas, circularity as represented by the isoperimetric ratio, and the radial distance for each cluster. Localization density is shown in gray. For all selected clusters, the convex hull region is shown in light blue and the centroid in red. It must be noted that the cluster selection does not represent a specific kind of cluster but only serves as objective identification procedure for a heterogeneous cluster set that contains larger ring-like structures. Scaling various throughout the panels; bin size = 20 nm.

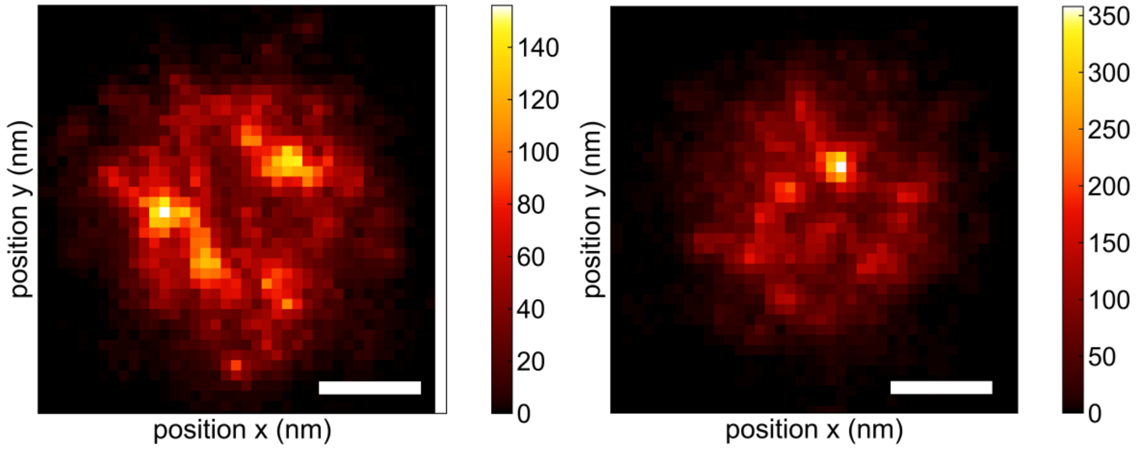

Supplementary Fig. 23. Overlay images of localization clusters for Munc13-1 and RIM signals. Clusters were determined on the combined Munc13-1 and RIM signals by DBSCAN and selected based on convex hull area and circularity (as described and shown in Fig. S22), shifted by their centroid position and rebinned (bin size=20 nm) as overlay figure. The spatial distribution indicates the variety of substructures hiding the center hole that clearly appears in selected clusters. Color scale represents localizations per pixel. Scale bar, 200 nm (7.5x expanded dimensions).

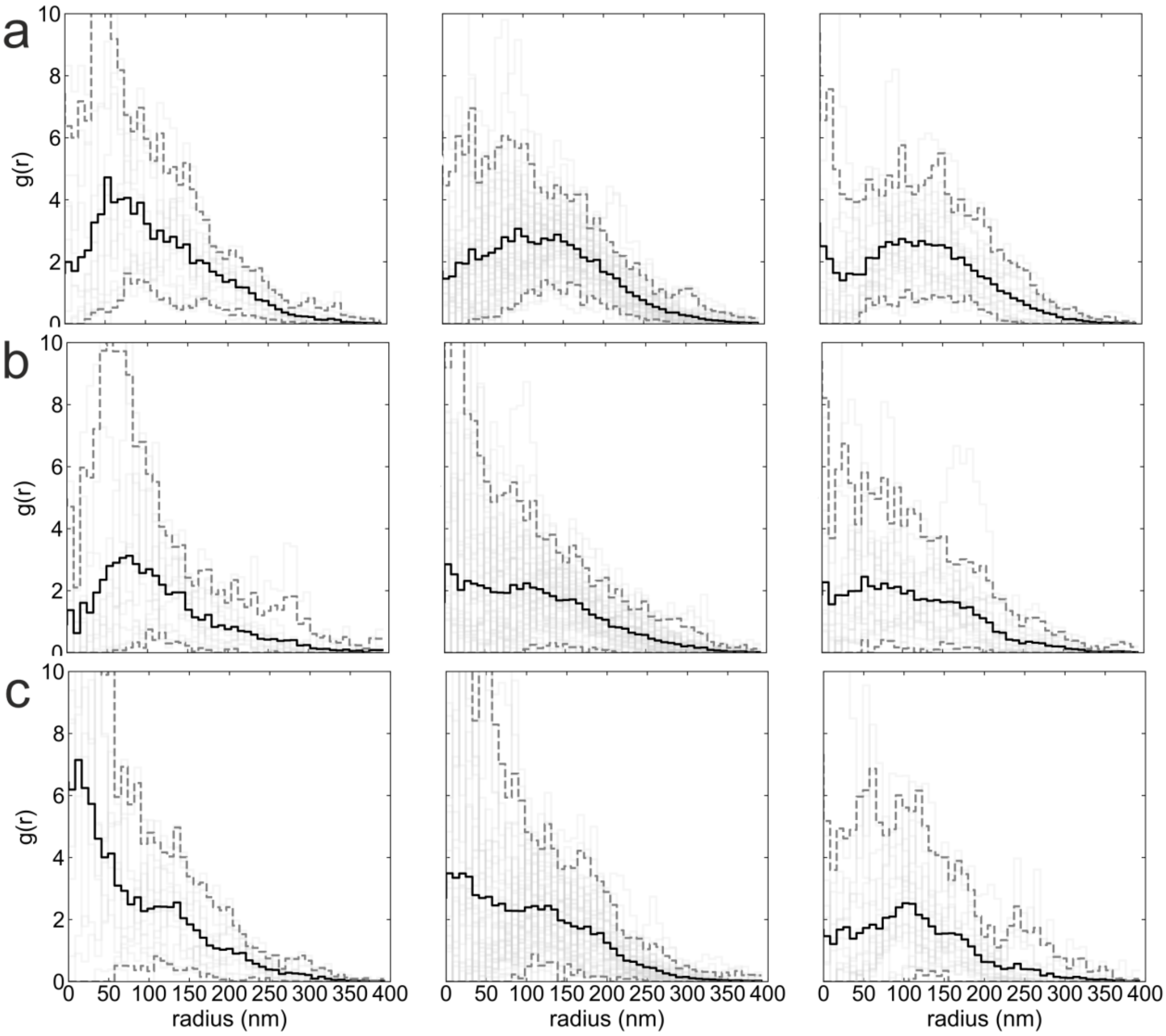

Supplementary Fig. 24. Radial distribution function for clusters of Munc13-1 and RIM signals. The radial distribution function is shown for all distances between localizations in Munc13-1 and RIM clusters and the cluster centroid in expanded samples: **a,** The radial distribution function for the combined Munc13-1 and RIM signals. **b,** The radial distribution function for Munc13-1 signals. **c,** The radial distribution function for RIM signals. In all panels data is shown for treatments DMSO-control (left, n=10), PMA stimulated (center, n=22) and untreated (right, n=16). Radial distribution functions are plotted (light gray lines) together with mean (black line) and 5/95 % confidence intervals (dashed gray lines). Scales represent 7.5x expanded dimensions.

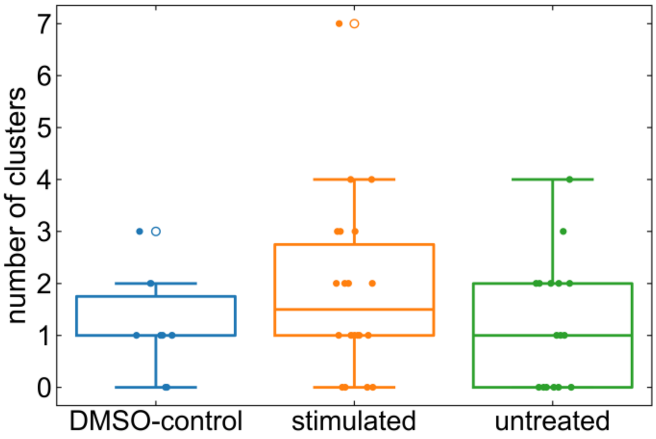

Supplementary Fig. 25. Number of clusters of Munc13-1 and RIM signals per region of interest. Clusters were determined as described and shown in Supplementary Fig. 22. The mean number of clusters were not significantly different between the various treatments DMSO-control (left, n=10), PMA stimulated (center, n=22) and untreated (right, n=16).

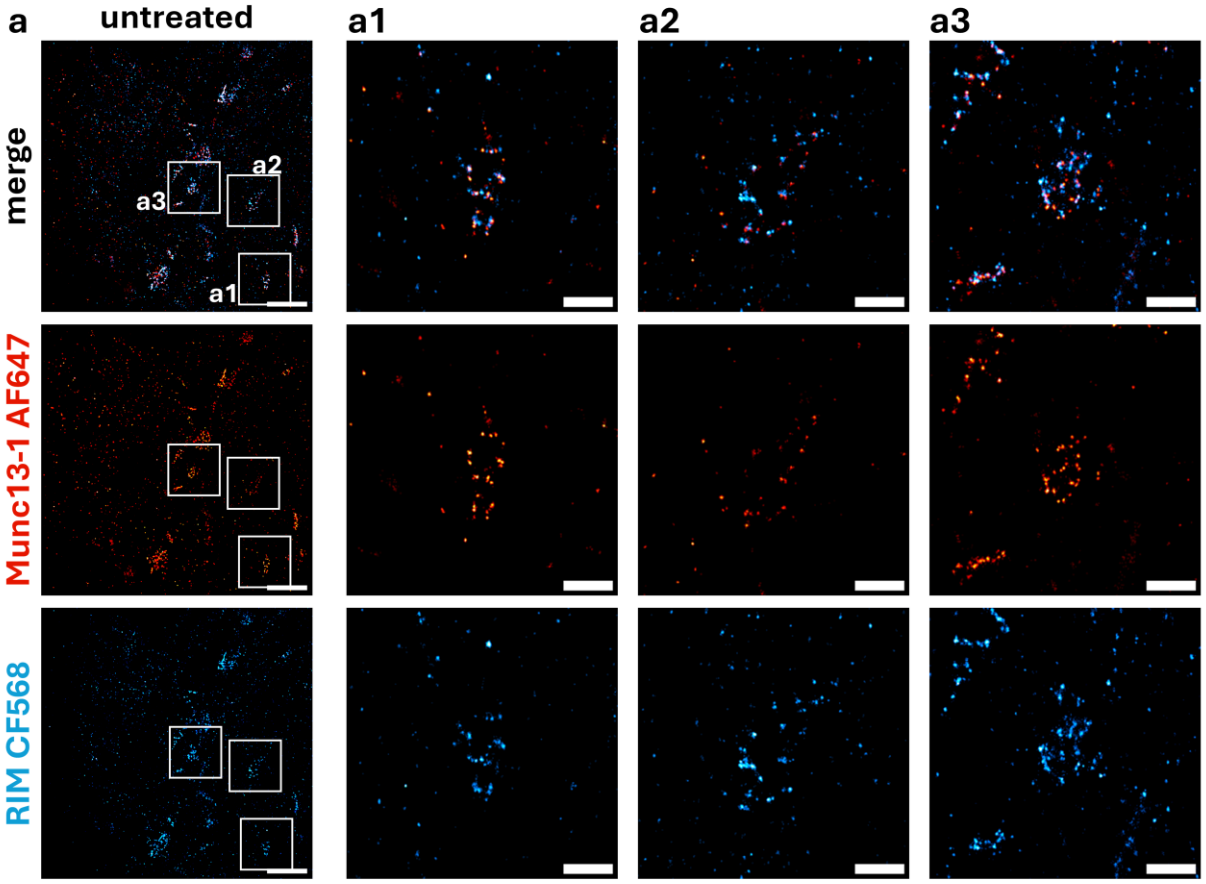

Supplementary Fig. 26. Two-color Ex-*d*STORM images of Munc13-1 and RIM1/2 using only denaturation during expansion. Untreated hippocampal mouse neurons were processed with a single TREx hydrogel using FA fixation, FA/AA anchoring and denaturation with SDS and DTT at 98°C and no proteinase K digestion. Substructures lack details and seem not properly expanded. **a**, Representative overview image. Selected frontal views of synapses are marked by a white square and magnified in a1, a2 and a3. Scale bars, a, 5 µm; a1-a3, 1 µm. Pixel size a, 60 nm; a1-a3, 20 nm. Scale bars show expanded dimensions with an estimated expansion factor of 5-6 (single TREx gel shrinks ~20 % during re-embedding in the neutral hydrogel).
