# Supplementary material for: Resolving endogenous protein organization in cells with nanometer resolution
Source: Nat Commun. 2026 Jul 29;17:7583. doi: 10.1038/s41467-026-76146-7 (PMC13421506; doi:10.1038/s41467-026-76146-7)
Supplement: Supplementary file 3 — Transparent Peer Review File [file 41467_2026_76146_MOESM3_ESM.pdf]

nature portfolio

Peer Review File

Resolving endogenous protein organization in cells with nanometer resolution

Corresponding Author: Professor Markus Sauer

This manuscript has been previously reviewed at another journal. This document only contains information relating to versions considered at Nature Communications.

This file contains all reviewer reports in order by version, followed by all author rebuttals in order by version.

Version 0:

Reviewer comments:

Reviewer #1

(Remarks to the Author)
The authors have largely addressed my prior comments, and I believe the manuscript is suitable for publication in Nature Communications pending a few final clarifications outlined below.

1) The authors have softened their claims regarding directly observing the 8 nm microtubule periodicity, which I believe is appropriate. However, I'm still a bit confused about how the 37.5 nm linkage error contributes to the amplitude of the 12 nm peak. If I understand correctly, the linkage error represents an offset introduced by the antibody at an intermediate expansion step in the protocol and the 37.5 nm is the error is what's computed after final expansion? So, scaling back down to the original (non-expanded) reference frame, the error would be 37.5 nm / (3X 2nd expansion scale) = 12.5 nm? If this is correct, then more explicitly stating this in text would be helpful for the reader.

Please also add these details to the "Simulation" methods section which currently only states an uncertainty of 2 nm (xy) and 5 nm (z). I'm assuming that the new simulation now incorporates an additional 12.5 nm uncertainty from linkage error that is added in quadrature? Please clarify in the final publication.

Reviewer #2

(Remarks to the Author)
I would like to thank the authors for their careful work and for addressing all of my previous comments. I have only one remaining point regarding the description of the microtubule structure.

In my opinion, the term "microtubule lattice" is too strong in this context and should be toned down. A more appropriate description would be that individual tubulin subunits (or molecules) within the microtubule can be visualized. The term lattice implies that the highly ordered cylindrical arrangement of tubulin is resolved, including features such as the distinction between the A-lattice and B-lattice. I do not believe that the data presented support this level of structural interpretation. Therefore, I suggest revising the wording accordingly.

Other than this minor point, I strongly recommend this article for publication.

Open Access This Peer Review File is licensed under a Creative Commons Attribution 4.0 International License, which permits use, sharing, adaptation, distribution and reproduction in any medium or format, as long as you give appropriate credit to the original author(s) and the source, provide a link to the Creative Commons license, and indicate if changes were made.
In cases where reviewers are anonymous, credit should be given to 'Anonymous Referee' and the source.
The images or other third party material in this Peer Review File are included in the article's Creative Commons license, unless indicated otherwise in a credit line to the material. If material is not included in the article's Creative Commons license and your intended use is not permitted by statutory regulation or exceeds the permitted use, you will need to obtain permission directly from the copyright holder.
To view a copy of this license, visit https://creativecommons.org/licenses/by/4.0/

Point-by-point response to the reviewers' comments

We wish to thank the Reviewers for their positive feedback. We have revised the manuscript in depth taking the final remarks into account. Reviewer comments are shown here in black, while our responses are indicated in blue text.

Reviewer #1 (Remarks to the Author):

The authors have largely addressed my prior comments, and I believe the manuscript is suitable for publication in Nature Communications pending a few final clarifications outlined below.

We thank the reviewer for his/her positive assessment.

1) The authors have softened their claims regarding directly observing the 8 nm microtubule periodicity, which I believe is appropriate. However, I'm still a bit confused about how the 37.5 nm linkage error contributes to the amplitude of the 12 nm peak. If I understand correctly, the linkage error represents an offset introduced by the antibody at an intermediate expansion step in the protocol and the 37.5 nm is the error is what's computed after final expansion? So, scaling back down to the original (non-expanded) reference frame, the error would be 37.5 nm / (3X 2nd expansion scale) = 12.5 nm? If this is correct, then more explicitly stating this in text would be helpful for the reader.

We thank the reviewer for this reference and added a more detailed explanation in the text:

"Taking into account experimental uncertainties (localization, antibody orientation etc.), the linkage error of ~37.5 nm (or ~4.7 nm unexpanded) translates into an axial offset of the measured positions close to half the lattice periodicity. Axial distances at multiples of half the lattice periodicity appear as higher harmonics at 32 (4) nm (twice the spatial frequency) in the axial autocorrelation, overlaying the 64 (8) nm periodicity of the tubulin positions."

Experimental evidence for this explanation would require systematic variation of the linkage error. Therefore, we can only speculate. An alternative explanation for the appearance of the 12 nm peak is a twist of the microtubule seam. Further studies will be required in the future to better understand the origin of the higher harmonics in the autocorrelation.

Please also add these details to the "Simulation" methods section which currently only states an uncertainty of 2 nm (xy) and 5 nm (z). I'm assuming that the new simulation now incorporates an additional 12.5 nm uncertainty from linkage error that

is added in quadrature? Please clarify in the final publication.

We updated the description of the simulations in the Methods part of the final version of the manuscript as requested by the reviewer:

“An additional offset of 37.5 nm (3 x 12.5 nm) in a random direction due to the linkage error after the second expansion and a localization uncertainty of 2 nm (xy) and 5 nm (z) (unexpanded) was used to define the final positions of the detected cluster centers.”

Reviewer #2 (Remarks to the Author):

I would like to thank the authors for their careful work and for addressing all of my previous comments. I have only one remaining point regarding the description of the microtubule structure.

We thank the reviewer for his/her positive assessment.

In my opinion, the term "microtubule lattice" is too strong in this context and should be toned down. A more appropriate description would be that individual tubulin subunits (or molecules) within the microtubule can be visualized. The term lattice implies that the highly ordered cylindrical arrangement of tubulin is resolved, including features such as the distinction between the A-lattice and B-lattice. I do not believe that the data presented support this level of structural interpretation. Therefore, I suggest revising the wording accordingly.

We thank the reviewer for careful reading. We changed the wording in the main text accordingly.

Other than this minor point, I strongly recommend this article for publication.
